# Insulin treatment improves liver histopathology and decreases expression of inflammatory and fibrogenic genes in a hyperglycemic, dyslipidemic hamster model of NAFLD

**DOI:** 10.1186/s12967-021-02729-1

**Published:** 2021-02-17

**Authors:** Victoria Svop Jensen, Christian Fledelius, Christina Zachodnik, Jesper Damgaard, Helle Nygaard, Kristina Steinicke Tornqvist, Rikke Kaae Kirk, Birgitte Martine Viuff, Erik Max Wulff, Jens Lykkesfeldt, Henning Hvid

**Affiliations:** 1 Section of Experimental Animal Models, Department of Veterinary and Animal Sciences, Faculty of Health and Medical Sciences, University of Copenhagen, Ridebanevej 9, 1870 Frederiksberg, Denmark; 2 Diabetes Pharmacology, Novo Nordisk A/S, Novo Nordisk Park 1, 2760 Måløv, Denmark; 3 Pathology & Imaging, Novo Nordisk A/S, Novo Nordisk Park 1, 2760 Måløv, Denmark; 4 Gubra ApS, Hørsholm Kongevej 11B, 2970 Hørsholm, Denmark

**Keywords:** NAFLD, NASH, Non-alcoholic fatty liver disease, Non-alcoholic steatohepatitis, Animal models, Hamster, Histopathology, Gene expression, Insulin treatment, Insulin therapy, Diabetes

## Abstract

**Background:**

Non-alcoholic fatty liver disease (NAFLD) and non-alcoholic steatohepatitis (NASH) are highly prevalent comorbidities in patients with Type 2 diabetes. While many of these patients eventually will need treatment with insulin, little is known about the effects of insulin treatment on histopathological parameters and hepatic gene expression in diabetic patients with co-existing NAFLD and NASH. To investigate this further, we evaluated the effects of insulin treatment in NASH diet-fed hamsters with streptozotocin (STZ) -induced hyperglycemia.

**Methods:**

Forty male Syrian hamsters were randomized into four groups (n = 10/group) receiving either a NASH-inducing (high fat, fructose and cholesterol) or control diet (CTRL) for four weeks, after which they were treated with STZ or sham-injected and from week five treated with either vehicle (CTRL, NASH, NASH-STZ) or human insulin (NASH-STZ-HI) for four weeks by continuous s.c. infusion via osmotic minipumps.

**Results:**

NASH-STZ hamsters displayed pronounced hyperglycemia, dyslipidemia and more severe liver pathology compared to both CTRL and NASH groups. Insulin treatment attenuated dyslipidemia in NASH-STZ-HI hamsters and liver pathology was considerably improved compared to the NASH-STZ group, with prevention/reversal of hepatic steatosis, hepatic inflammation and stellate cell activation. In addition, expression of inflammatory and fibrotic genes was decreased compared to the NASH-STZ group.

**Conclusions:**

These results suggest that hyperglycemia is important for development of inflammation and profibrotic processes in the liver, and that insulin administration has beneficial effects on liver pathology and expression of genes related to inflammation and fibrosis in a hyperglycemic, dyslipidemic hamster model of NAFLD.

## Background

The prevalence of non-alcoholic fatty liver disease (NAFLD) as well as its more progressed form non-alcoholic steatohepatitis (NASH) is increasing world-wide [1] and is in the general population estimated to be approximately 25% and 1.5–6.5%, respectively [2]. However, in patients with Type 2 diabetes mellitus (T2DM), the prevalence of NAFLD and NASH diagnosed by imaging modalities was recently estimated to be 55.5% and 37.3%, respectively [3]. T2DM is one of the most important risk factors for developing NAFLD and NASH. It is a predictor of progressive liver disease, as well as both overall and liver-related mortality [3–6]. Many patients with T2DM will eventually need insulin treatment but whether exogenous insulin accelerates or attenuates development of or co-existing NAFLD and NASH is not known. A hypothetical adverse effect of insulin treatment on progression of NAFLD would be concerning, as NAFLD has been associated with an increased incidence of cardiovascular disease [7, 8] (although causality remains controversial [9, 10]), which accounts for the majority of deaths in T2DM [11].

Previous clinical trials where hepatic effects of insulin therapy in diabetic patients were explored, focused on hepatic fat content and non-specific circulating markers of the disease (e.g., aminotransferases) with no evaluation of liver histology and hence on progressive liver disease [12–19]. Insulin is known to stimulate hepatic de novo lipogenesis and might therefore be expected to increase hepatic lipid content. Nonetheless, the above-mentioned studies collectively indicate that conventional insulin treatment results in a decrease in hepatic fat content, with the largest effect observed in the initial phases of treatment [12–19], likely due to insulin-mediated inhibition of lipolysis in the adipose tissue, resulting in reduced flux of free fatty acids (FFA) to the liver [20]. A reduction in hepatic fat content could potentially exert beneficial effects on e.g. hepatic inflammation and fibrosis, however, some retrospective studies have indicated an association between insulin therapy in diabetes and increased risk of hepatic fibrosis and hepatocellular cancer [21–23]. Whether these findings are causally linked to the insulin therapy per se or due to other factors is not known. The effects of insulin treatment on hepatic parameters in diabetic animal models of NAFLD/NASH have been examined in a few preclinical studies and these studies report varying effects of insulin treatment on hepatic inflammation and fibrosis [24–27]. Therefore, the effects of insulin treatment on the development and progression of NAFLD in a diabetic setting remain unclear. Furthermore, the previous studies were performed in mouse or rat models. It is well described that there are important differences between human lipid metabolism and that of mice and rats [28–30], which could complicate the interpretation and compromise the translational value of results from mouse and rat models in which NAFLD/NASH is induced by lipid-enriched diets.

The aim of this study was to investigate hepatic effects of insulin therapy in a NASH diet- and streptozotocin (STZ)-induced hamster model of diabetic NAFLD. The hamster was chosen as a model, because of the similarity in plasma lipid and lipoprotein metabolism compared to humans [30–32]. Our hypotheses were that (i) hyperglycemia accelerates liver pathology compared to effects of a high-fat diet alone and that (ii) insulin therapy improves liver pathology by preventing or reversing hepatic steatosis, inflammation and fibrosis. Using RNA-sequencing (RNAseq), we furthermore investigated global hepatic expression patterns in the model and compared it to gene expression patterns in human NAFLD, to explore the translational potential of the hamster model.

## Materials and methods

### Animals

Forty male Syrian hamsters were procured from Janvier Labs (Le Genest-Saint-Isle, France) at the age of six or seven weeks at arrival and weighing ~ 80–100 g. The hamsters were housed four animals/cage and were acclimatized for a week prior to initiation of the study. During the acclimatization period the animals had unrestricted access to standard rodent chow (1324 Altromin, Brogaarden, Denmark), and non-chlorinated, non-acidified tap water. Temperature in the animal rooms was set between 20 and 25 °C, with a light/dark cycle of 12/12 h, a relative humidity of 30–70% and air changes 8–15 times/h. The study was approved by the Danish Animal Experiments Inspectorate in accordance with European Union Directive 2010/63/EU.

### Experimental design

**Fig. 1:**
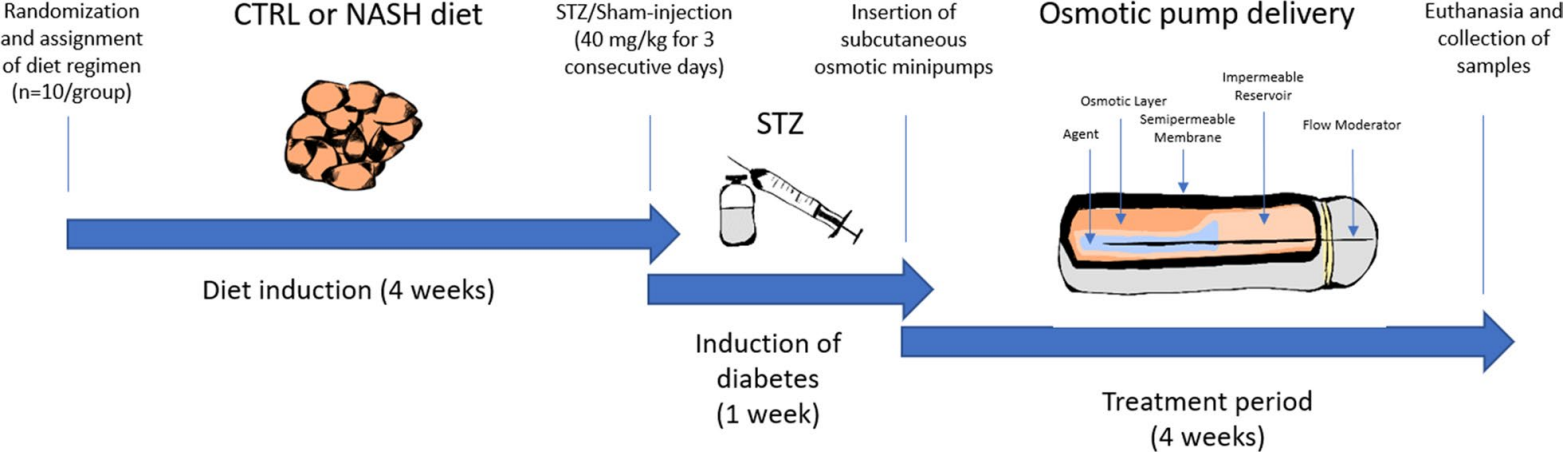
Overview of study design. Animals were assigned to receive either a control diet (one group, n = 10) or a NASH-diet (three groups, n = 10/group) for four weeks. After four weeks, two of the NASH-diet-fed groups (NASH-STZ, and NASH-STZ-HI) were injected subcutaneously with STZ (40 mg/kg) on three consecutive days, while the control-diet group (CTRL) and the remaining NASH-diet-group (NASH) were sham-injected with saline. One week after STZ/Sham-injections, osmotic minipumps containing either vehicle (CTRL, NASH, NASH-STZ) or Human Insulin (NASH-STZ-HI) were surgically inserted subcutaneously. After a four-week treatment period the animals were euthanized, and tissue samples were collected. STZ: Streptozotocin

After the acclimatization period, animals were block randomized based on body weight (restricted by cages of four) into four groups (n = 10/group), and then randomly assigned to receive either a purified control diet (D16010104, Research Diets, NJ, US, one group, Fig. 1) or a high-fat/high-fructose/high-cholesterol NASH diet (D16010102, Research Diets, NJ, US, three groups, Fig. 1). Ten animals per group were estimated to be sufficient to obtain reliable estimates of mean value and variability of the blood glucose lowering effect of human insulin. After four weeks on diets, two of the groups receiving the NASH diet were randomly assigned to be injected subcutaneously with 40 mg/kg STZ (Sigma-Aldrich, Soeborg, Denmark) suspended in 0.1 M sodium citrate buffer (pH 4.5) in the morning for three consecutive days (Fig. 1). The group receiving the control diet (CTRL) and the remaining NASH diet-fed group (NASH) were injected s.c. with the sodium citrate buffer at the same time points. One week after the STZ/sodium citrate buffer injections, blood glucose and HbA1c was measured by tail vein sampling in all animals. In addition, animals were weighed and scanned using quantitative magnetic resonance (qMR). To ensure comparable blood glucose and body weight levels, all STZ-injected animals were stratified by blood glucose and body weight into two new groups intended to receive either vehicle-treatment (NASH-STZ, n = 10) or treatment with human insulin (NASH-STZ-HI, n = 10). The hamsters were first treated with 5 mg/kg Rimadyl (Bela-Pharm GmbH, Vechta, Germany) and 5 mg/kg Baytril (KVP Pharma + Veterinär Produkte GmbH, Kiel, Germany) by s.c. injection. Then they were anaesthetized in isoflurane (Baxter, IL, USA) and a small transverse incision of approximately 1 cm was made in the subcutis between the shoulder blades. By blunt dissection, a pouch of approximately 1 × 5 cm was made in the subcutis, extending caudally from the incision site. An Alzet osmotic minipump (model: 2ML4, ALZET Osmotic Pumps, CA, US) containing either vehicle (CTRL, NASH, NASH-STZ) or human insulin (NASH-STZ-HI, dose: 40 nmol/kg/day) was then inserted in the pouch. The incision site was subsequently closed using surgical clips (Becton–Dickinson, MD, US). Following the surgical procedure, the animals were treated once daily for two days with 5 mg/kg Rimadyl (5 mg/kg). The s.c. osmotic minipumps remained implanted for four weeks (Fig. 1), during which blood glucose was monitored once a week. After four weeks of treatment a large blood sample was collected from the sublingual vein for evaluation of plasma parameters, and the animals were qMR-scanned. The animals were euthanized by exsanguination while in deep isoflurane anaesthesia. Immediately thereafter, the liver was excised and weighed, and samples were collected from the middle section of the left lateral lobe, the right medial lobe and the caudal lobe and transferred to 10% neutral buffered formalin (Hounisen Laboratorieudstyr A/S, Skanderborg, Denmark) for histopathological analysis. Another sample was collected from the right medial lobe and snap-frozen for cryo-sectioning. Six punch biopsies (diameter: 4 mm/biopsy) were then collected from the left lateral lobe and snap-frozen in liquid nitrogen for later RNA-extraction and biochemical analyses. Lastly, the epididymal fat depots (left and right) were excised and weighed.

### Euthanized/excluded animals

From the STZ-injection period (week five) until termination of the study (week nine), three animals from the NASH-STZ group were euthanized due to severe hyperglycemia. Furthermore, one animal from the CTRL group was euthanized before the treatment period due to the presence of an abscess (incidental finding). Finally, one animal from the NASH-STZ-HI group was euthanized after insertion of the osmotic minipumps, due to weight loss, and failure to recover completely from the surgical procedure.

### Body weight and body composition

Body weight was monitored once weekly throughout the study period. For quantification of changes in total fat mass and lean mass, animals were qMR-scanned on the day of pump insertion (treatment start) and again at study termination, using an EchoMRI Body Composition Analyser (EchoMRI, Houston, TX, USA) as described previously [33].

### Blood and plasma samples

Blood samples for assessment of blood glucose and HbA1c were collected from the tail vein in heparin-coated capillary tubes (Vitrex Medical, Herlev, Denmark) once weekly throughout the treatment period. Blood glucose was assessed with the glucose oxidase method at a Biosen Apparatus (EKF Diagnostics, Barleben, Germany) according to manufacturer’s instructions. HbA1c was determined using a Cobas 6000 c501 instrument (Roche Diagnostics GmbH, Mannheim, Germany) according to manufacturer’s instructions. In addition, larger blood samples were collected into K_3_-EDTA microvette tubes (Sarstedt AG & Co, Nümbrecht, Germany) from the sublingual vein in conscious, non-fasted animals on the morning of the day of euthanization. After centrifugation, plasma was isolated and kept at − 20 °C until further analysis. Quantification of very-low-density lipoprotein triglyceride (VLDL-TG), low-density lipoprotein cholesterol (LDL-C), high-density-lipoprotein cholesterol (HDL-C) and total cholesterol in plasma was done using gel-filtration high performance liquid chromatography at LipoSEARCH (Skylight Biotech Inc, Akita, Japan). Plasma levels of free fatty acids (FFA), haptoglobin, alanine aminotransferase (ALT) and aspartate aminotransferase (AST) were measured using a Cobas 6000 c501 instrument (Roche Diagnostics GmbH, 68206 Mannheim, Germany) according to the manufacturer’s instructions. Plasma concentration of endogenous hamster insulin was measured as described previously with an assay designed for detection of rat insulin [34]. The results are therefore presented as “rat insulin immunoactivity equivalents” (RIIE) as the cross reactivity to hamster insulin was not determined. In samples where equivalents were below the lower limit of quantification (LLOQ, 20 pM), these were set to be LLOQ/2 (10 pM) for subsequent statistical analysis, as described previously [35]. Plasma concentration of human insulin was measured in the NASH-STZ-HI group as described previously [36]. The LLOQ for the human insulin assay was 15 pM.

### Liver biochemistry

To determine hepatic levels of triglyceride (TG), total cholesterol (TC) and glycogen, liver biopsies from the left lateral lobe were homogenized in a 0.15 M sodium acetate buffer (pH = 4.9) containing 0.75% Triton-X100 (Sigma-Aldrich, Soeborg, Denmark), placed on a heating block (90–100 °C) for two min and subsequently cooled on ice. For each sample the cooled homogenate was then split into two aliquots. Amyloglucosidase (Sigma Aldrich) was added to one of the aliquots, which was then placed on a heating block (50 °C) for 2 h to facilitate the breakdown of glycogen to glucose. Both aliquots were subsequently centrifuged at 9000*g* for 10 min and the supernatants were analyzed using a Cobas 6000 c501 instrument (Roche Diagnostics GmbH, Mannheim, Germany) according to manufacturer’s instructions.

### Liver histology

Sections of liver from the left lateral, right medial and caudal lobe were embedded in paraffin [37]. Sections of 3 µm thickness were then stained with Mayer’s Haematoxylin and Eosin (H&E, Sigma-Aldrich). Cryo-sections were stained with Oil Red O (Sigma-Aldrich) to evaluate and confirm the presence of hepatic steatosis. Presence of hepatocyte ballooning was evaluated on H&E stains. Hepatic inflammation was visualized using immunohistochemistry (IHC) to detect CD68-positive (CD68+), CD11b-positive (CD11b+) and CD45-positive (CD45+) cells. Details on the different IHC protocols are shown in Additional file 1: Table S1. For a small subset of animals, a double-staining of CD68 (IHC) and Oil Red O was performed on cryo-sections from the left lateral liver lobe, to characterize the content of CD68+ cells. Collagen deposition and hepatic stellate cell activation was evaluated using histochemical staining with Picro Sirius Red (Sigma-Aldrich) and IHC for α-SMA (Additional file 1: Table S1), respectively. All sections were scanned using a NanoZoomer 2.0 HT slide scanner (Hamamatsu, Hamamatsu City, Japan) and subsequently evaluated using NanoZoomer Digital Pathology Image Software (Hamamatsu). Image analysis for quantification of inflammation (CD68, CD11b, CD45), collagen deposition (Picro Sirius Red) and hepatic stellate cell activation (α-SMA) was performed with VIS software (Visiopharm, Hoersholm, Denmark). With automated threshold analysis applications, the area with positive staining for each marker of interest was identified in each tissue section and expressed as a percentage of the total area of the tissue section (i.e., the fractional area). For each marker of interest, the same threshold for identification of positive staining was used across all tissue sections in the study.

### Extraction of RNA from liver samples and RNA-sequencing

Total RNA was extracted from homogenized liver samples using a RNeasy Mini Kit (Qiagen, Aarhus, Denmark) according to the manufacturer’s instructions. The RNA integrity number (RIN) of each RNA samples was determined with a Bioanalyzer 2100 (Agilent Technologies, CA, US). RNA samples from five animals per group was sent to Qiagen (Aarhus, Denmark) for RNASeq. Average RIN-value of samples sent to Qiagen was 8.3 with SD of 0.4. At Qiagen, library preparation was done using a TruSeq^®^ Stranded mRNA Sample preparation kit (Illumina, CA, US). A starting material of 500 ng of total RNA was mRNA-enriched using the oligodT-bead system and subsequently fragmented by enzymatic treatment. Thereafter, first- and second strand synthesis was performed, and the resulting double stranded cDNA was purified (AMPure XP, Beckman Coulter, IN, US). The cDNA was end repaired, 3′-adenylated and Illumina sequencing adaptors were ligated onto the fragment ends, after which the library was purified (AMPure XP, Beckman Coulter). The libraries size distribution was validated and the subjected to quality control using a 2200 TapeStation system (Agilent Technologies, CA, US). High quality libraries were pooled based in equimolar concentrations based on the Bioanalyzer Smear Analysis tool (Agilent Technologies). The library pool(s) were quantified using quantitative PCR and optimal concentration of the library pool was used to generate clusters on the surface of a flow cell, before sequencing on a NextSeq 500 instrument (75 cycles) according to the manufacturer’s instructions (Illumina). An average of 37 million reads were obtained for each sample. Raw reads obtained from the Illumina pipeline were mapped back to the Golden Hamster reference genome (Ensembl_93/MesAur1.0). The raw read counts were used in R 3.5.1 [38] for data preprocessing. All non-protein coding genes as well as protein-coding genes expressed in less than 25% of the samples were filtered out. In total 11,061 of the 35,020 genes passed this filter and were analysed as described below.

### Differential gene expression analysis

Differential gene expression analysis was performed using Qlucore Omics Explorer 3.0 software (Qlucore AB, Lund, Sweden) and Metacore software (Version 19.4) from Clarivate Analytics. Genes and pathways with a Benjamini-Hochberg-adjusted p-value < 0.05 (corresponding to a 5% False Discovery Rate) and fold changes below − 1.5 or above + 1.5 were considered statistically significantly regulated.

### Comparison to human NASH data sets

The hamster gene expression dataset was compared to two human gene expression data sets [39, 40]. From the study by Suppli et al. [39] a list of differentially expressed genes between patients with histologically proven hepatic steatosis and mild inflammation (NAFL) and patients with NASH (characterized by steatosis, inflammation and fibrosis) was extracted and used to generate a heat map of these genes visualizing differences in gene expression between NASH and NASH-STZ hamsters. Furthermore, the authors of this study had defined a panel of 112 so-called “candidate NAFLD genes”, involved in e.g. insulin signaling, monocyte recruitment, inflammation signaling, hepatocellular cell death and stellate cell activation [39]. This panel of genes was probed on the hamster data set, to explore differences in expression of the NAFLD candidate genes between the NASH and the NASH-STZ group. Data from the NAFL and NASH patient groups in the study by Lake et al. [40] were imported into Qlucore, and analyzed with thresholds similar to the analysis of the hamster RNAseq data (i.e., q-value < 0.05 and fold-change > 1.5-fold). The resulting list of differentially expressed genes between human NASH and NAFL patients was compared to the list of differentially expressed genes between NASH-STZ and NASH hamsters, and genes regulated in both species were plotted on heatmaps, to visualize the gene expression patterns.

### Validation of RNA Sequencing results with quantitative real-time PCR

RNA extracted from homogenized liver tissue was used to synthesize cDNA using an iScript cDNA Synthesis Kit (BioRad Laboratories, Denmark) according to the manufacturer’s instructions. Eight genes were selected from the RNA sequencing analysis results for validation by quantitative real-time PCR (qRTPCR), chosen both from lists of top 10 up- and downregulated genes (*Gpnmb, Pklr, Spp1, Soat1*, Table 1) or from genes represented in significantly enriched pathways (*Ccl2, Il1b, Col3a1, Timp1,* Additional file 2: Table S2). Expression of each target gene was normalized to the average expression of the two reference genes *Hprt1* and *Rpl18* using the delta–delta Ct method. *Hprt1* and *Rpl18* both displayed stable expression across experimental groups. Primer sequences for the eight validation genes and the two reference genes are shown in Table 2 and were designed using the free online software Primer3^®^ (Whitehead Institute for Biomedical Research, Cambridge, MA 02142, United States). The qRT-PCR was performed using the SYBR™ Green method [41]. In brief, 8 µL of Mastermix (containing 1 µL primer mix (5 µM of forward primer and 5 µM of reverse primer), 2 µL RNAse-free water and 5 µL PowerUp™ SYBR™ Green Master Mix) and 2 µL sample cDNA was transferred to a 96-well plate. For negative controls, RNAse-free water was added to wells instead of sample. The qPCR-reaction was carried out on samples (in triplicates) in an Applied Biosystems real-time-PCR thermal cycler (StepOnePlus™ Real-Time PCR System) under the following conditions: 50 °C for 2 min, 95 °C for 5 min and 40 cycles of 95 °C for 10 s, 60 °C for 10 s and 72 °C for 20 s. Melting curves were produced and evaluated for all primer sets. The data were analyzed using StepOne Software vers. 2.3. (Applied Biosystems, CA, US).

**Table 1:** Top 10 regulated genes (up/down) by diet, hyperglycemia and treatment

Gene symbol	Gene name	q-value	log2-fold change NASH vs CTRL
Upregulated genes			
*Grid2ip*	Glutamate receptor, ionotropic, delta-2 interacting protein-1	1.42 × E−04	4.29
*Htra3*	Htra serine peptidase-3	6.51 × E−04	3.80
*Wscd2*	WSC domain containing 2	6.51 × E−04	3.75
*Kcnip3*	Potassium voltage-gated channel interacting protein-3	4.50 × E−04	3.66
*Gpx6*	Glutathione peroxidase-6	3.23 × E−04	3.35
*Fabp3*	Fatty-acid binding protein 3	5.21 × E−04	3.30
*Cxcl10*	CXC motif chemokine ligand 10	2.27 × E−03	2.84
*Anxa2*	Annexin A2	3.46 × E−03	2.48
*Tuba8*	Tubulin alpha 8	1.86 × E−04	2.46
*Ubd*	Ubiquitin D	1.06 × E−03	2.42
Downregulated genes			
*Lipg*	Lipase G	9.35 × E−04	− 3.08
*Cd163*	Macrophage-associated antigen	1.28 × E−03	− 2.52
*Sqle*	Squalene epoxidase	2.45 × E−03	− 2.46
*Pcsk9*	Proprotein convertase subtilisin/kexin type 9	3.78 × E−04	− 2.39
*Cish*	Cytokine inducible SH2 containing protein	1.65 × E−03	− 2.17
*Btg2*	B-Cell translocation gene	2.85 × E−03	− 2.12
*Trim24*	Tripartite motif containing 24	1.06 × E−03	− 1.83
*Aldh1a3*	Aldehyde dehydrogenase 1 family member A3	2.28 × E−03	− 1.66
*Fgb*	Fibrinogen beta chain	1.78 × E−0.3	− 1.59
*Fgg*	Fibrinogen gamma chain	1.62 × E−0.3	− 1.58

Gene symbol	Gene name	q-value	log2-fold change NASH-STZ vs NASH
Upregulated genes			
*Gpnmb*	Transmembrane glycoprotein NMB	2.00 × E−03	5.43
*Spp1*	Secreted phosphoprotein 1	2.14 × E−03	4.31
*Tff3*	Trefoil factor 3	1.29 × E−03	4.27
*Clec4d*	C-Type lectin domain family 4 member D	1.39 × E−03	3.81
*Chrna1*	Cholinergic receptor nicotinic alpha 1 subunit	8.25 × E−04	3.73
*Tm4sf19*	Transmembrane 4 L six family member 19	1.28 × E−03	3.68
*Soat1*	Sterol O acyltransferase-1	1.02 × E−03	3.64
*Acp5*	Tartrate-resistant acid phosphatase 5	9.67 × E−04	3.49
*Ctsh*	Cathepsin H	9.08 × E−04	3.46
*Clec5a*	C-type lectin domain family 5 member A	9.76 × E−04	3.42
Downregulated genes			
*Hcn3*	Hyperpolarization activated cyclic nucleotide gated potassium channel 3	3.71 × E−03	− 4.38
*Serpina5*	Serpin family A member 5	3.49 × E−03	− 4.19
*Pnpla3*	Patatin-like phospholipase domain containing 3	2.71 × E−03	− 4.13
*Gck*	Glucokinase	2.13 × E−03	− 3.90
*Pklr*	Pyruvate kinase L/R	3.10 × E−03	− 3.35
*Mogat2*	Monoacylglycerol O-Acyltransferase 2	1.28 × E−03	− 3.02
*Gp5*	Glycoprotein V platelet	2.27 × E−03	− 2.91
*Cpxm2*	Carboxypeptidase X, M14 family member 2	4.33 × E−03	− 2.80
*Aadacl4*	Arylacetamide deacetylase-like 4	6.03 × E−03	− 2.80
*Acly*	ATP citrate lyase	8.95 × E−04	− 2.72

Gene symbol	Gene name	q-value	log2-fold change NASH-STZ-HI vs NASH-STZ
Upregulated genes			
*Hcn3*	Hyperpolarization activated cyclic nucleotide gated potassium channel 3	4.48 × E−03	4.25
*Serpina5*	Serpine family A member 5	3.81 × E−03	4.14
*Pnpla3*	Patatin-like phospholipase domain containing 3	4.74 × E−03	3.65
*Gck*	Glucokinase	4.44 × E−03	3.57
*Pklr*	Pyruvate kinase L/R	3.65 × E−03	3.25
*Gp5*	Glycoprotein V platelet	3.72 × E−03	2.61
*Abhd5*	Abhydrolase domain containing-5	1.71 × E−03	2.12
*Mogat2*	Monoacylglycerol O-Acyltransferase 2	2.18 × E−03	2.09
*Me1*	NADP-dependent malic enzyme	5.97 × E−03	1.89
*Ppp1r3b*	Protein phosphatase 1 regulatory subunit 3B	3.33 × E−03	1.77
Downregulated genes			
*Tff3*	Trefoil factor 3	3.75 × E−03	− 3.42
*Spp1*	Secreted phosphoprotein 1	3.77 × E−03	− 3.01
*S100g*	S100 calcium binding protein G	6.28 × E−03	− 2.92
*Gpnmb*	Transmembrane glycoprotein NMB	2.96 × E−03	− 2.92
*Soat1*	Sterol O-Acyltransferase 1	2.28 × E−03	− 2.84
*Clec4d*	C-type lectin domain family 4 Member D	6.78 × E−03	− 2.59
*Bst1*	Bone marrow stromal cell antigen 1	3.41 × E−03	− 2.59
*Slc16a6*	Solute carrier family 16 member 6	3.88 × E−03	− 2.58
*Rpb2*	RNA polymerase II gene	2.27 × E−03	− 2.56
*Fst*	Follistatin	2.27 × E−03	− 2.54

**Table 2:** Primer sequences

Gene ID	Forward primer	Reverse primer
*Gpnmb*	TTGCGCAAGTGAAAGACATC	CCAGTGTTGTCCCCAAAGTT
*Pklr*	TGACCTAGTTACCCGCAACC	CAGGGCGTCTTAGAATCCAG
*Spp1*	ACAGCCAGGATTCTGTGGAC	TTTGCCCGTAGTCCATAAGC
*Soat1*	GAGCAAGATCAAGCCCAGAG	TCGTCGAGAATGGAGAAGGT
*Ccl2*	CAGAGAGACTCAGGCCAACC	AGCAGGTGAGTGGGGAGTTA
*Il*-*1β*	GCAGGTGGTGTCAGTCATTG	AGACAGCACGAGGCATTTCT
*Col3a1*	CTTTGTGCAAAGTGGGACCT	TCTCCAAATGGGATCTCTGG
*Timp*-*1*	TCCCTTGCAAACTGGAGAGT	GGGTAGGCTTTGGGTCATTT
Housekeeping genes		
*Hprt1*	TGATCAGTCAACAGGGGACA	AGAGGTCCTTTTCACCAGCA
*Rpl18*	AGAACAAGACTGCCGTGGTT	GGTATACCTCTCGGCCCTTC

### Statistics

For all data (except RNAseq data) statistical analyses and graphs were done using GraphPad Prism version 8.02 (GraphPad Software Inc., La Jolla, CA, US). Data were assessed for normal distribution and variance homology by visual inspection of qq-plots and plots of residuals versus predicted values, respectively. Data which were not normal distributed or with heterogeneous variance were transformed by the natural logarithm. If meeting the above-mentioned assumptions, differences in means between groups were analyzed using one-way ANOVA followed by Tukey’s post hoc tests for multiple pairwise comparisons between groups. Post hoc tests were performed only when the level of F reached statistical significance (*p *< 0.05). For data not meeting the assumptions required for the use of an ANOVA, a non-parametric Kruskal–Wallis test with subsequent Dunn test for multiple comparisons was used. *p*-values < 0.05 were considered statistically significant. Data are presented as mean values with SEM in figures and mean values with SD in tables.

## Results

### Insulin treatment increased body weight, fat mass and lean mass in NASH-STZ hamsters

**Fig. 2:**
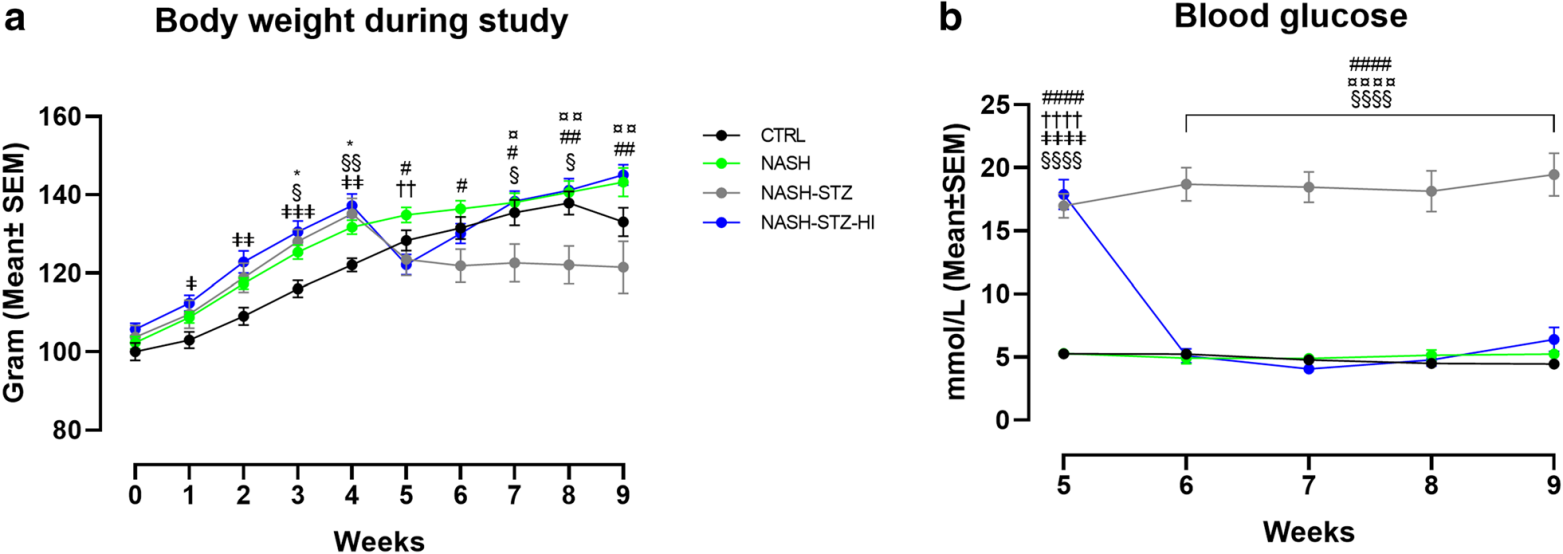
Insulin treatment lowered blood glucose and increased body weight, fat mass and lean mass in NASH-STZ hamsters. Development in **a** body weight during the nine-week study period and **b** blood glucose during the four-week treatment-period in CTRL, NASH, NASH-STZ and NASH-STZ-HI. ^*^ indicate *p *< 0.05 between CTRL and NASH; ^#,^^##,^^####^ indicate *p *< 0.05, *p *< 0.01 and *p *< 0.0001 between NASH and NASH-STZ; ^¤, ¤¤,^^¤¤¤¤^ indicate *p *< 0.05, *p *< 0.01 and *p *< 0.0001 between NASH-STZ and NASH-STZ-HI; ^§,^^§§^ and ^§§§§^ indicate *p *< 0.05, *p *< 0.01 and *p *< 0.0001 between CTRL and NASH-STZ; ^ǂ,^ ^ǂǂ,^ ^ǂǂǂ^ and ^ǂǂǂǂ^ indicate *p *< 0.05, *p *< 0.01, *p *< 0.001 and *p *< 0.0001 between CTRL and NASH-STZ-HI; ^††^ and ^††††^ indicate *p *< 0.01 and *p *< 0.0001 between NASH and NASH-STZ-HI

The influence of diet, hyperglycemia and insulin treatment on body composition is outlined in Fig. 2 and Table 3. The body weight (BW) of NASH-fed hamsters was higher compared to those fed CTRL diet during the diet-induction period (*p *< 0.05 or less, Fig. 2), but was not significantly different for the remainder of the study. From the start of the STZ-injection period until the start of the insulin treatment period (week five), the NASH-STZ and NASH-STZ-HI groups lost ~ 10% of their pre-STZ BW. During the insulin treatment period (week 5-9), the insulin-treated NASH-STZ-HI group increased in BW compared to NASH-STZ (Fig. 2) and was higher compared to the NASH-STZ group from week seven until study termination (*p *< 0.05 or less). Fat mass during the treatment period increased only in the insulin-treated group, where the change in fat mass was higher compared to that of the CTRL and the NASH-STZ groups at study termination (*p *< 0.0001, Table 3). Lean mass increased during the treatment period in all groups, and the change in lean mass in the NASH-STZ-HI group was higher compared to that of the NASH-STZ group at study termination (*p *< 0.05, Table 3). The epididymal fat depots collected at euthanasia weighed more in NASH and NASH-STZ-HI groups compared to the CTRL and NASH-STZ groups (*p *< 0.001 or less).

**Table 3:** Metabolic parameters at euthanization

Metabolic parameters	CTRL	NASH	NASH-STZ	NASH-STZ-HI
ΔFat mass (termination-treatment start) (g)^c^	− 3.9 (± 3.7)^†^	− 0.2 (± 6.18)	− 5.6 (± 3.3)^†^	7.7 (± 3.4)^*,¤^
ΔLean mass (termination-treatment start) (g)	12.3 (± 9.5)	10.7 (± 7.9)	6.3 (± 9.0)^†^	19.1 (± 3.5)^¤^
Epididymal fat depot (g)	1.4 (± 0.3)^#,†^	2.6 (± 0.5)^*^	1.1 (± 0.4)^#^	2.3 (± 0.4)^*,¤^
Liver weights (g/100g body weight)	2.9 (± 0.2)^¤,†^	5.3 (± 0.4)^*,†^	5.5 (± 0.4)^*,†^	4.6 (± 0.5)^*,#,¤^
Liver TG (µmol/g)^a^	7.4 (± 1.9)^#,¤^	14.4 (± 4.5)^*,¤^	48.1 (± 16.4)^*,#,†^	14.3 (± 4.8)^*,¤^
Liver cholesterol (µmol/g)^a^	5.6 (± 1.4)^#,¤,†^	70.9 (± 14.3)^*^	72.8 (± 13.2)^*^	87.3 (± 14.9)^*^
Liver glycogen (µmol/g)	245.6 (± 145.4)^¤^	372.3 (± 93.8)	56.5 (± 94.8)^*,#,†^	300.9 (± 51.5)^¤^
Plasma 3-β-hydroxybutyrate (µmol/L)	406.5(± 257.2)^¤^	464.3(± 89.8)^¤^	3750(± 2150)^*,#,†^	451.8(± 148.5)^¤^
ΔHbA1c (%)^c^	− 0.2 (± 0.2)^¤^	− 0.1 (± 0.2)	2.0 (± 0.7)^*,†^	− 0.6 (± 0.4)^¤^
Endogenous insulin (RIIE)^a^	265.5 (± 295.2)	472.3 (± 406.5)^¤^	97.7 (± 110.4)^#^	NA
Exposure HI (pM)	NA	NA	NA	246.5 (± 163.1)
Plasma free fatty acids^a^ (mmol/L)	0.7 (± 0.5)^¤^	0.8 (± 0.5)^¤^	2.0 (± 1.3)^*,#^	1.0 (± 0.4)
Plasma VLDL-TG (mg/dL)^a^	134.1 (± 61.8)^#,¤,†^	256.9 (± 36.5)^*,¤^	1074.0 (± 389.1)^*,#,†^	247.0 (± 75.2)^*,¤^
Plasma total cholesterol (mg/dL)^a^	122.1 (± 18.3)	237.1 (± 25.1)^*^	405.7 (± 75.6)^*,#,†^	233.5 (± 19.0)^*,¤^
Plasma LDL-C (mg/dL)^a^	37.8 (± 22.9)^¤^	24.3 (± 3.4)^¤^	79.4 (± 29.0)^*,#,†^	28.6 (± 4.7)^¤^
Plasma HDL-C (mg/dL)	46.9 (± 22.3)^#,†^	130.7 (± 22.4)^*,¤^	50.5 (± 39.5)^#,†^	124.8 (± 9.3)^*,¤^
Alanine aminotransferase (ALAT)^b^ (U/L)	52.0 (± 21.5)	118.1 (± 53.3)	359.3 (± 250.0)	236.0 (± 88.5)
Aspartate aminotransferase (ASAT)^b^ (U/L)	50.0 (± 23.8)	51.4 (± 21.4)	109.1(± 46.8)	83.1 (± 47.9)
Plasma haptoglobin^a^ (g/L)	1.7 (± 1.4)^¤^	0.9 (± 0.3)^¤^	12.3 (± 6.0)^*,#,†^	0.9 (± 0.1)^¤^

HI: Human insulin; RIIE: Rat insulin immunoactivity equivalents; TG: triglyceride; VLDL: very-low-density lipoprotein; LDL-C: low-density lipoprotein cholesterol; HDL: high-density lipoprotein cholesterol

Results are presented as mean ± SD. Parameters denoted with ^a^ were ln-transformed before statistical analysis. Parameters denoted with ^b^ were not subjected to statistical analysis due to there being too few analysis results in the NASH-STZ group (n = 2–3). Parameters denoted with ^c^ were analysed by non-parametic statistical method (Kruskal–Wallis). Superscripts (^*,#,¤,†^) indicate significant differences (*p *< 0.05 or less) in readouts compared to CTRL-, NASH-, NASH-STZ- and NASH-STZ-HI-group respectively. Sample sizes: CTRL (n = 9), NASH (n = 10), NASH-STZ (n = 7), NASH-STZ-HI (n = 9)

### Pronounced hyperglycemia and dyslipidemia was seen in NASH-STZ hamsters and was partly reversed by insulin treatment

Plasma parameters for each group are shown in Table 3. Mean blood glucose was higher in STZ-injected groups compared to the CTRL- and NASH groups at treatment start (*p *< 0.0001, Fig. 2). Insulin treatment reduced mean blood glucose to normoglycemic levels in the NASH-STZ-HI group within the first week of treatment and remained stable and significantly lower compared to NASH-STZ for the remaining part of the study (*p *< 0.0001, Fig. 2). The increase in HbA1c during the study period was higher in NASH-STZ hamsters compared to CTRL and NASH hamsters (*p *< 0.01*)* and this increase was reversed by insulin treatment (*p *< 0.0001, Table 3). The levels of endogenous insulin (given as RIIE) were decreased in NASH-STZ hamsters compared to NASH (*p *< 0.05, Table 3) but was not significantly different from those of the CTRL group. Hyperglycemia resulted in pronounced dyslipidemia in the NASH-STZ group, characterized by increased plasma levels of FFA, VLDL-TG, total cholesterol and LDL-C compared to CTRL and NASH groups (*p *< 0.05 or less, Table 3) and decreased plasma HDL-C compared to the NASH group (*p *< 0.0001, Table 3). Insulin treatment significantly improved the dyslipidemia by lowering plasma levels of VLDL-TG, total cholesterol and LDL-C (*p *< 0.01 or less) and increasing plasma HDL-C (*p *< 0.0001, Table 3) compared to NASH-STZ. FFA was not significantly decreased by insulin treatment, but a trend towards 50% lower FFA levels in insulin-treated animals was observed. Assessment of ALT and AST in plasma samples from the NASH-STZ group proved difficult due to a high concentration of circulating plasma lipids. Consequently, statistical analysis was not performed on these parameters. However, the average levels of ALT and AST in plasma were numerically higher in the NASH-STZ-group compared to all other groups (Table 3). Plasma levels of 3-β-hydroxybutyrate was higher in NASH-STZ compared to both CTRL and NASH (*p *< 0.0001) and were decreased with insulin treatment (*p *< 0.0001, Table 3). Finally, levels of plasma haptoglobin were higher in NASH-STZ compared both CTRL and NASH (*p *< 0.0001) and were decreased with insulin treatment (*p *< 0.0001, Table 3).

### Liver histopathology in NASH-STZ hamsters resembled NAFLD and insulin treatment had beneficial effects on several histological parameters

**Fig. 3:**
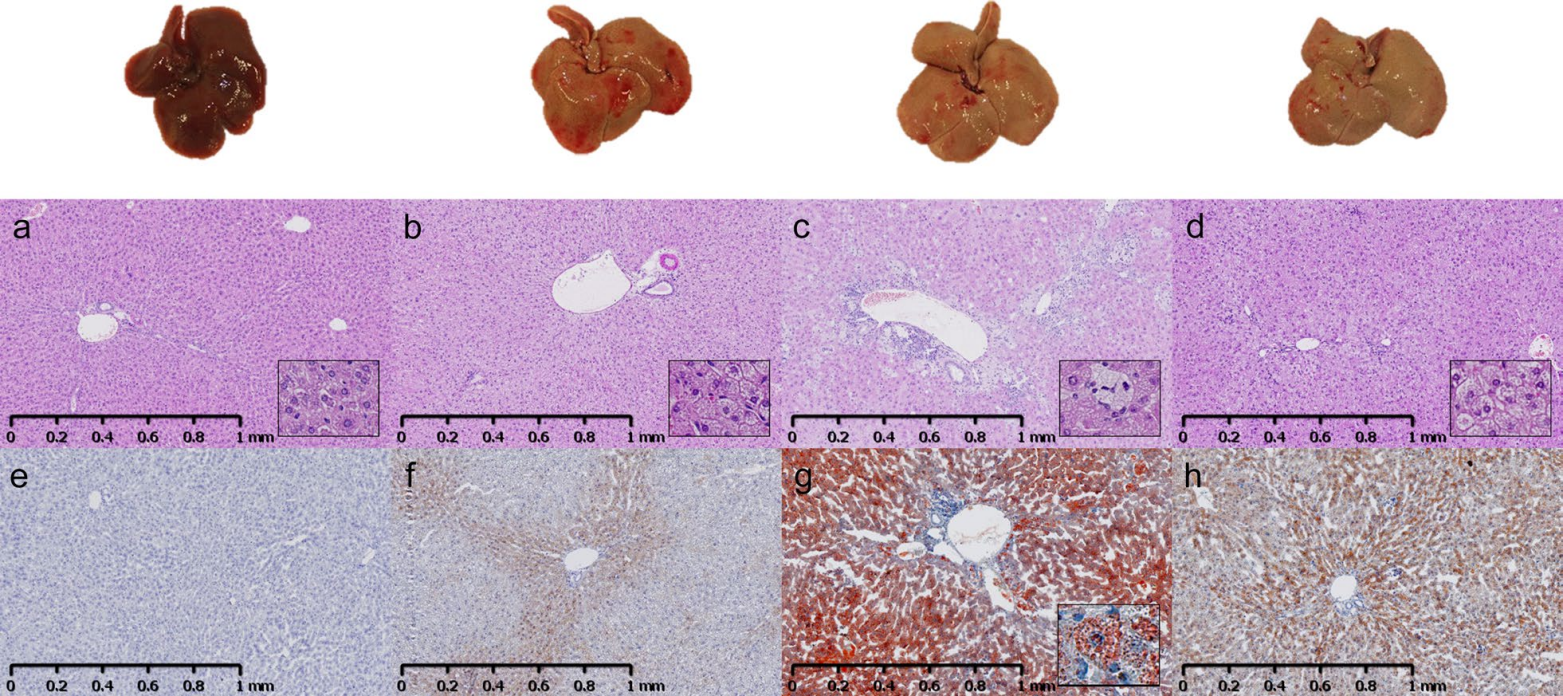
Liver histopathology in NASH-STZ hamsters resembled NAFLD and insulin treatment improved hepatic steatosis. Top row: Macroscopic appearance of the livers from CTRL-, NASH-, NASH-STZ- and NASH-STZ-HI immediately after euthanization (left to right). **a**–**d** Representative H&E stained sections of liver from CTRL-, NASH-, NASH-STZ- and NASH-STZ-HI hamsters (left to right). **e**–**h** Representative Oil Red O stained sections of liver from CTRL-, NASH-, NASH-STZ- and NASH-STZ-HI hamsters (left to right). Insert in **g**: ×20 magnification of lipid accumulation in hepatocyte. Inserts in **a**-**d**: x20 magnifications of H&E-stained sections

**Fig. 4:**
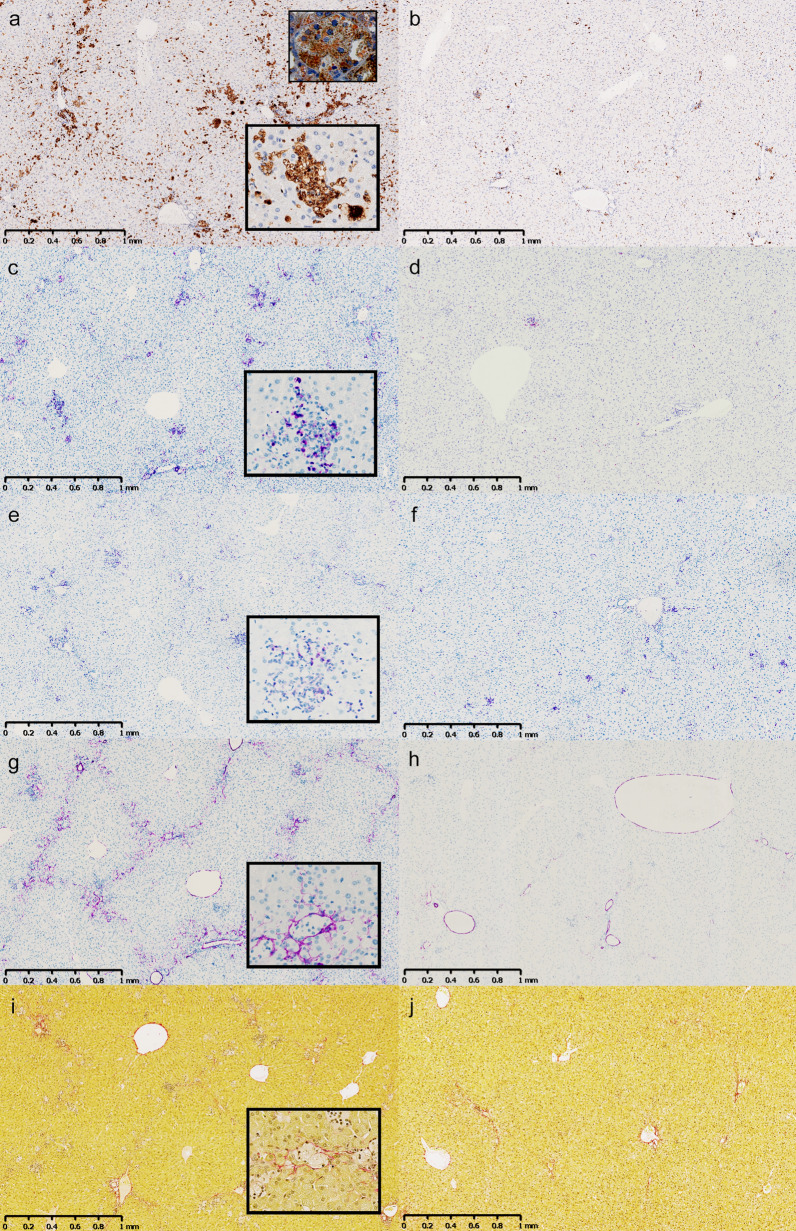
Hepatic inflammation and activation of stellate cells was induced in the NASH-STZ hamster and was prevented or reversed by insulin treatment. Representative sections of liver from the NASH-STZ group (**a**, **c**, **e**, **g**, **i**) and the NASH-STZ-HI group (**b**, **d**, **f**, **h**, **j**) displaying positive staining of markers CD68 (**a**, **b**), CD11b (**c**, **d**), CD45 (**e**, **f**), αSMA (**g**, **h**), Picro Sirius Red (**i**, **j**). Smallest insert in upper right corner of **a**: CD68 and Oil Red O double stain (x40 magnification). Inserts in **a**, **c**, **e**, **g** and **i**: x10 magnifications of positively stained cells/fibres

**Fig. 5:**
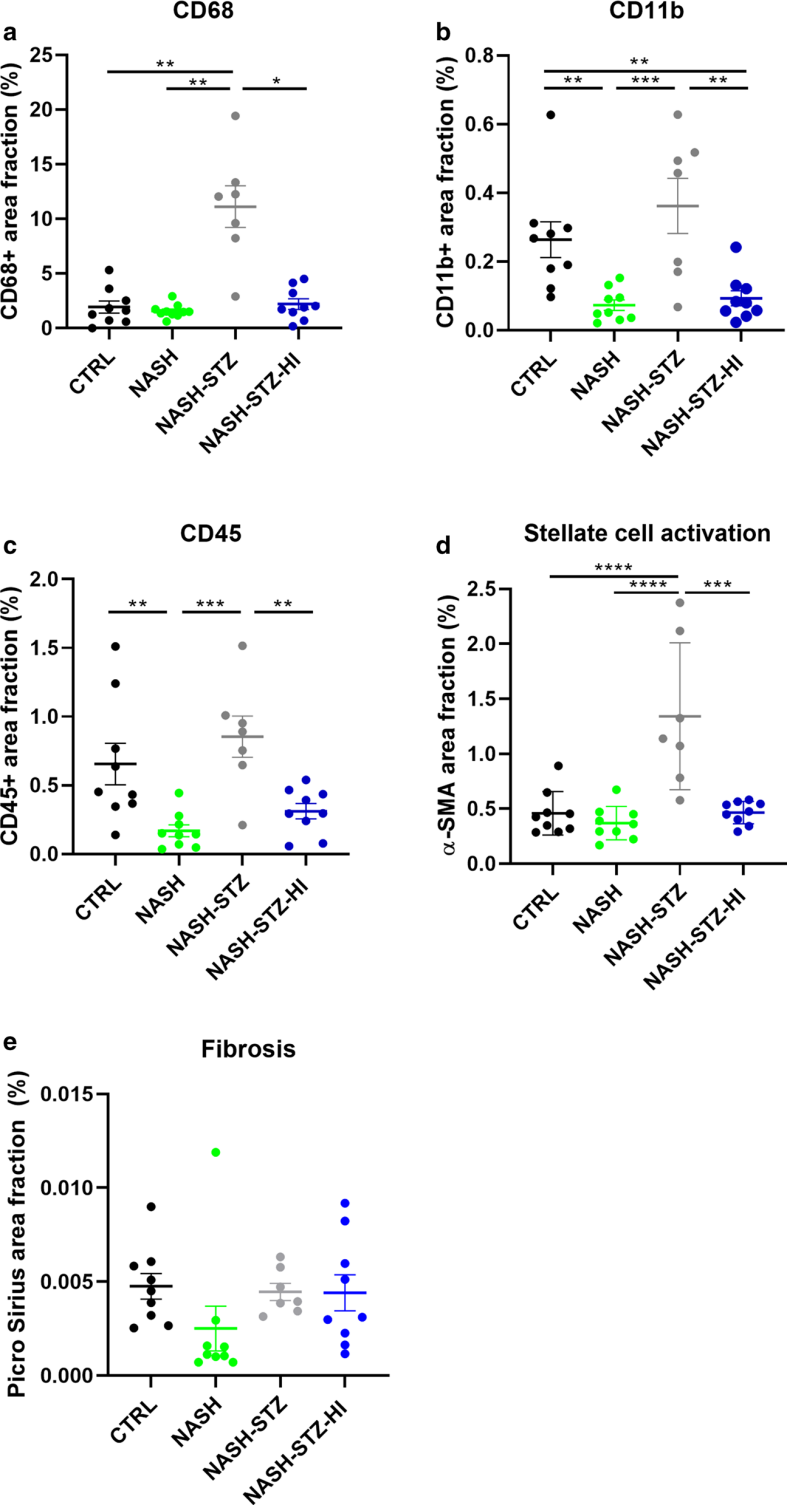
Image analysis revealed that insulin treatment prevented or reversed hepatic inflammation and stellate cell activation in the NASH-STZ hamster. Differences in area fractions of **a** CD68+ cells, **b** CD11b+ cells, **c** CD45+ cells, **d** αSMA, and **e** Picro Sirius Red in all groups

Representative macroscopic and histological images of the liver are shown in Figs. 3 and 4. Outputs from image analysis on IHC stains are shown in Fig. 5. Liver weights/100 g BW and hepatic biochemical parameters are shown in Table 3. H&E-stains revealed hepatic steatosis (primarily characterized by pin-point steatosis) in NASH, NASH-STZ and NASH-STZ-HI but not in CTRL (Fig. 3a–d). These findings were confirmed by Oil Red O stains (Fig. 3e–h), and biochemical parameters (hepatic triglyceride and cholesterol, Table 3). In the NASH-STZ animals, the steatosis was distributed throughout the entire liver parenchyma, whereas the steatosis in NASH and NASH-STZ-HI affected primarily zone 1 and 2 in the liver lobules. No ballooning hepatocytes were observed in any of the four groups. Image analysis showed a higher proportion of CD68+ cells in NASH-STZ livers compared to all other groups (*p *< 0.05 or less, Figs. 5a and 4a, b) and double-staining with CD68 and Oil Red O revealed that CD68 + cells contained lipid species (see smallest insert in Fig. 4a). Area fractions of CD45 + and CD11b+ cells were surprisingly lower in NASH-fed hamsters than CTRL hamsters (*p *< 0.01, Fig. 5b, c), but in NASH-STZ hamsters the CD45+ and CD11b+ area fractions were increased compared to NASH hamsters (*p *< 0.001). In insulin-treated animals both area fractions were significantly decreased compared to NASH-STZ hamsters (*p *< 0.01, Figs. 4c, e and 5b, c). There were no significant differences in hepatic collagen deposition between groups upon image analysis of Picro Sirius Red-stained sections (Figs. 4i, j and 5f), however, a higher fraction of α-SMA-positive (α-SMA+) cells (indicating activation of hepatic stellate cells [42]) were observed in livers of NASH-STZ animals compared to both CTRL and NASH (*p *< 0.0001, Fig. 5d) and was decreased after treatment with insulin (*p *< 0.001, Figs. 4g, h and 5d).

### Hepatic expression of inflammatory and fibrogenic genes was increased in NASH-STZ hamsters and decreased by insulin treatment

**Fig. 6:**
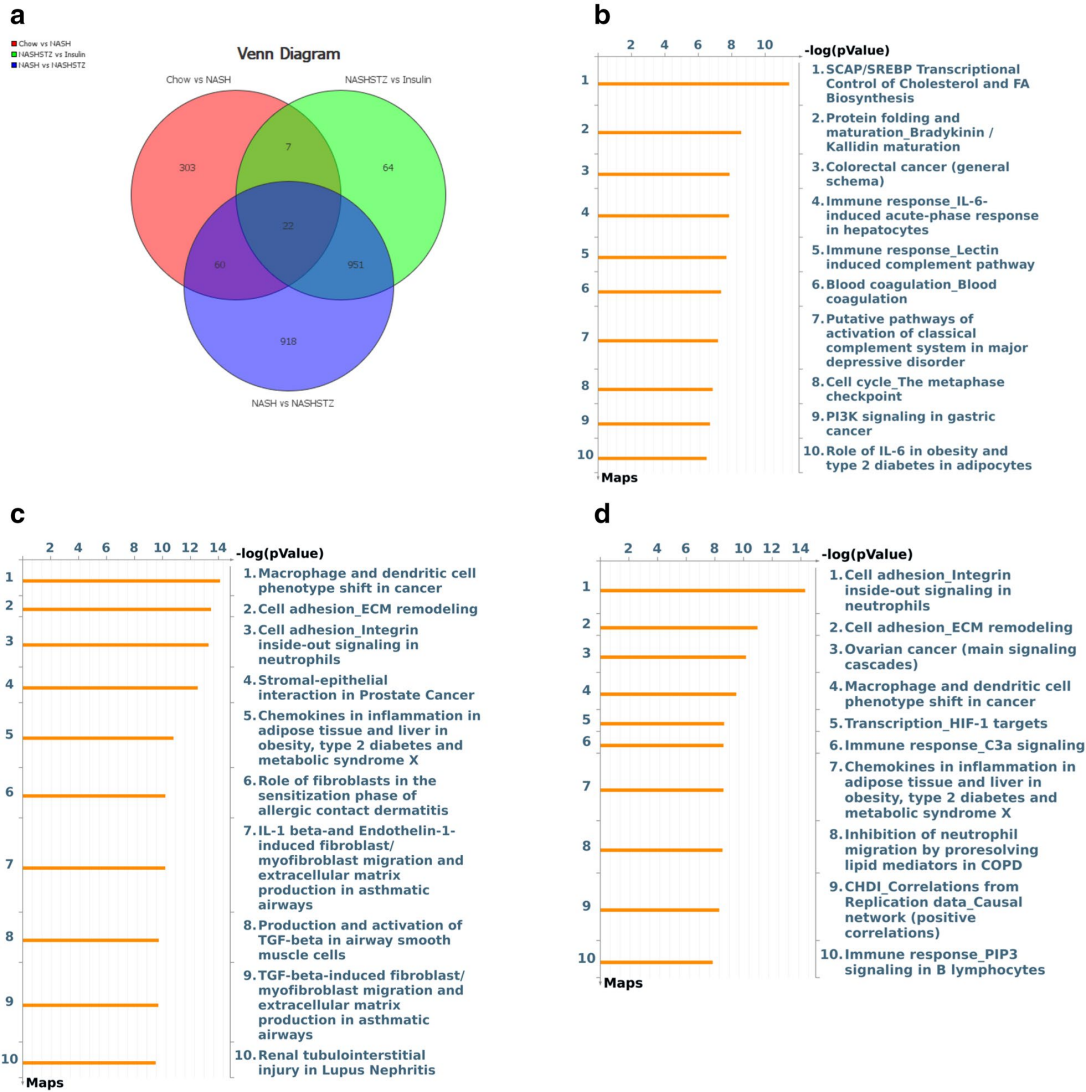

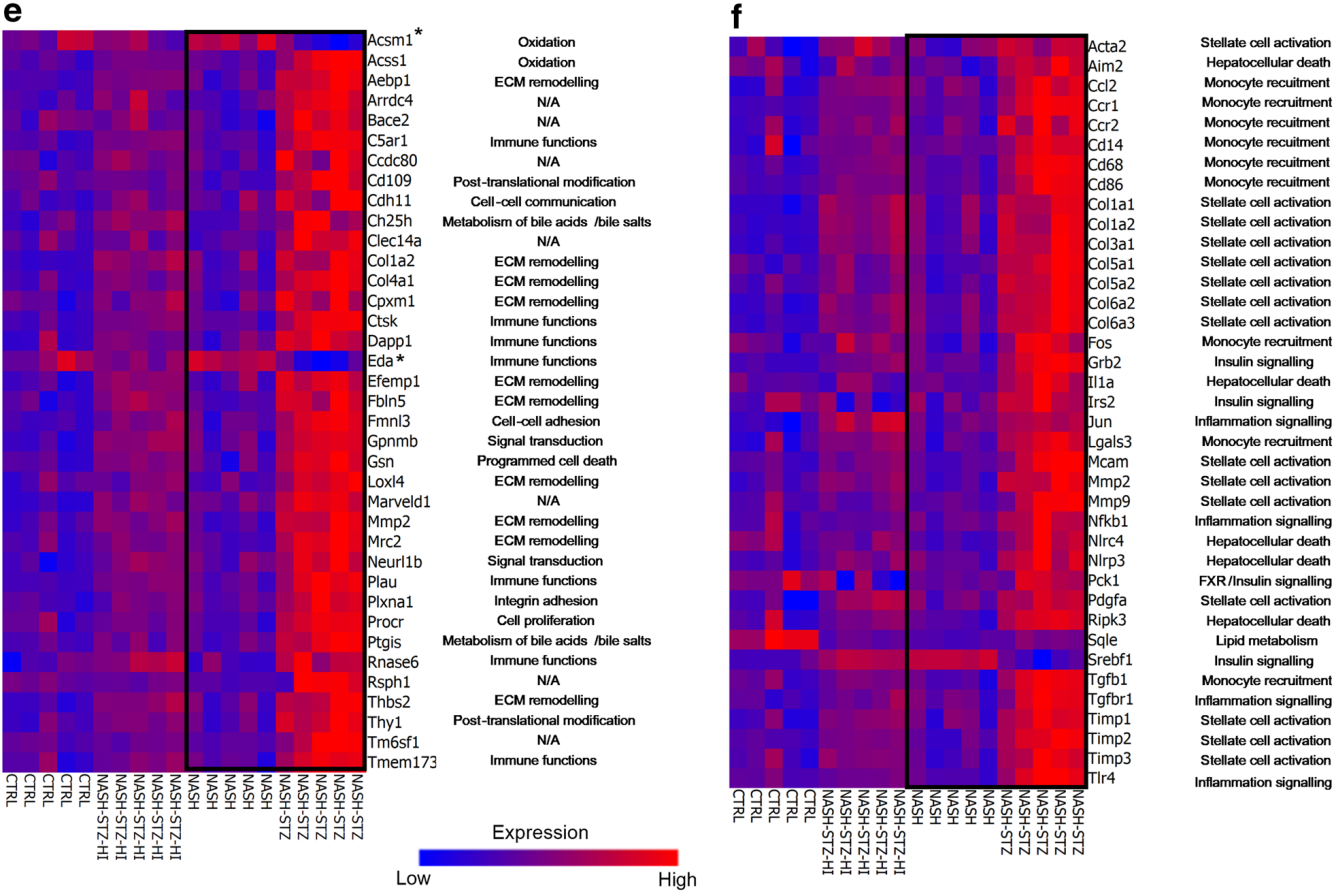
Pathways and genes related to proinflammatory and profibrotic processes were enriched/upregulated in the NASH-STZ, and this response was prevented or reversed by insulin treatment. **a** Venn Diagram of differentially expressed genes. **b**–**d** Pathway enrichment in the individual comparisons (diet (**b**), hyperglycemia (**c**), insulin treatment (**d**)). **e** Heat map based on reference list of differentially expressed genes in human patients with NASH compared to patients with NAFL [39], here visualizing regulation of the same genes in CTRL, NASH, NASH-STZ and NASH-STZ-HI. All genes except the ones marked with asterisks (*) are regulated similarly in NASH-STZ- vs. NASH-hamsters when compared to human NASH vs. NAFL. **f** Heat map showing regulation of a panel of NAFLD candidate genes taken from [39]. Upregulation of genes associated with e.g. monocyte recruitment, inflammation signalling, ECM remodelling and stellate cell activation are seen in the NASH-STZ hamsters compared to CTRL, NASH and NASH-STZ-HI

Venn-diagram visualizing regulation of genes due to the effect of diet (CTRL vs NASH), hyperglycemia (NASH vs NASH-STZ) and insulin treatment (NASH-STZ vs NASH-STZ-HI) are shown in Fig. 6a. Complete lists of genes significantly regulated by diet, hyperglycemia and insulin treatment are available in Additional file 1: Tables S3–S5. Enriched pathways are shown in Fig. 6b–d and pathway-associated regulated genes are shown in Additional file 2: Table S2. Pathways analysis revealed that diet (CTRL vs. NASH) regulated pathways associated with transcriptional control of cholesterol and fatty acid synthesis, protein folding and regulation of immune responses in hepatocytes (Fig. 6b and Additional file 2: Table S2). Hyperglycemia (NASH-STZ vs NASH) impacted regulation of pathways associated with inflammatory and fibrogenic responses in the liver such as “Macrophage and dendritic cell phenotypes”, “Chemokines in inflammation in adipose tissue and liver in obesity in type 2 diabetes and metabolic syndrome X” and “Cell adhesion and ECM remodeling” (Fig. 6c & Additional file 2: Table S2). The majority of the genes in these pathways were upregulated. Insulin treatment (NASH-STZ-HI vs NASH-STZ) impacted regulation of many of the same inflammation- and fibrosis-related pathways affected by hyperglycemia, however, with the majority of pathway-associated genes being downregulated (Fig. 6d). The top 10 significantly regulated genes (up- and down) associated with each comparison (diet, hyperglycemia, insulin treatment) are shown in Table 1.

### Gene expression data assessed with RNA sequencing were validated with quantitative RT-PCR

To validate the gene expression data based on RNAseq, eight genes were selected for validation using qRT-PCR (*Gpnmb, Soat1, Spp1, Pklr, Ccl2, Il1β, Col3a1* and *Timp1)*. Expression data for these eight genes assessed with qRT-PCR are shown in Table 4 and showed very good agreement between qRT-PCR and RNAseq data.

**Table 4:** qPCR-validation of selected genes from RNASeq

Gene symbol	NASH-STZ vs NASH				NASH-STZ-HI vs NASH-STZ			
	RNASeq (log2-fold change)	*q*-value	qPCR (log2-fold change)	*q*-value	RNASeq (log2-fold change)	*q*-value	qPCR (log2-fold change)	*q*-value
*Gpnmb*	5.43	2.00 × E−03	5.55	2.94 × E−05	− 2.92	2.96 × E−03	− 2.65	1.43 × E−02
*Pklr*	− 3.35	3.10 × E−03	− 3.58	3.22 × E−04	3.25	3.65 × E−03	3.55	3.48 × E−04
*Spp1*	4.31	2.14 × E−03	4.74	1.01 × E−05	− 3.01	3.77 × E−03	− 3.04	9.20 × E−04
*Soat1*	3.64	1.02 × E−03	3.62	1.71 × E−06	− 2.84	2.28 × E−03	− 2.50	1.14 × E−04
*Ccl2*	2.78	4.76 × E−03	3.19	1.25 × E−03	− 1.96	7.58 × E−03	− 1.72	5.10 × E−02
*Il1β*	1.72	< 0.05	1.54	4.16 × E−03	− 1.21	< 0.05	− 0.82	9.31 × E−02
*Col3a1*	1.64	1.93 × E−03	1.70	7.17 × E−05	− 0.96	5.06 × E−03	− 0.94	9.69 × E−03
*Timp1*	2.71	3.20 × E−03	2.69	3.13 × E−04	− 1.69	2.18 × E−03	− 1.57	1.70 × E−02

### The liver transcriptome in NASH-STZ hamsters resembled the transcriptome in human NAFLD/NASH

Heat maps visualizing hamster liver expression of genes which in two previous studies were found to be differently expressed between human patients with NAFL and NASH [39, 40] are shown in Fig. 6e and Additional file 3: Figure S1, respectively. 132 genes were found to be differently expressed (*q *< 0.05) between human NAFL and NASH in the study by Suppli et al. [39]. Of the 132 genes, 89 genes were identified in the hamster gene expression data set, and 37 out of these 89 genes were differently expressed by more than ± 1.5 fold when comparing NASH-STZ to NASH animals (Fig. 6e). Thirty-five of these 37 genes (95%) were similarly regulated in the NASH-STZ vs. NASH group when compared to human NASH vs. NAFL (Fig. 6e). Among others, these were genes involved in ECM remodeling, cell adhesion, immune functions, signal transduction and apoptosis (Fig. 6e). Regulation of the 37 genes in the insulin-treated animals clearly differed from that of the NASH-STZ-group, showing a greater resemblance to the transcriptomic signature of the NASH-fed group (Fig. 6e). The candidate NAFLD gene panel of 112 genes from [39] was probed on the hamster data set and 96 of these genes were present in the hamster data set. Of these, 38 genes were differently expressed by more than ± 1.5 fold between NASH and NASH-STZ hamsters (Fig. 6f). The 38 candidate genes were involved in monocyte recruitment, hepatocellular death, inflammation signaling, insulin signaling, lipid metabolism or stellate cell activation (Fig. 6f). In the second human data set ([40]), 887 genes were found to be differentially expressed by more than 1.5-fold (q < 0.05) between human patients with NAFL and NASH and of those 635 genes were present in the hamster data set. With a cut-off value of minimum ± 1.5-fold difference in expression, 209 genes were identified. Heat maps of the 209 differentially expressed genes in the human comparison [40] and in hamsters are seen in Additional file 3: Figure S1. Here, 91% (190/209) genes showed similar expression patterns in human NASH vs NAFL and hamster NASH-STZ vs NASH group. In the human data sets 35 genes were present in both [39] and [40] and differently expressed by more than ± 1.5 fold in the comparison of NAFL vs NASH. Sixteen of these genes were also present in the hamster data set and expressed by more than ± 1.5 fold when NASH-STZ and NASH was compared (Additional file 1: Table S6). These genes were involved in functions such as ECM remodeling, immune functions, programmed cell death, signal transduction, cell–cell communication, metabolism of bile acids and post-translational modification.

## Discussion

In this study, we investigated the effects of STZ-induced hyperglycemia on development and progression of NAFLD in hamsters, as well as the effects of insulin treatment on liver pathology and hepatic gene expression in a dyslipidemic, hyperglycemic setting. We found that induction of hyperglycemia exacerbated pathological progression of NAFLD in the hamster when superimposed on a NASH diet. Thus, hyperglycemia per se accelerated dyslipidemia, hepatic and systemic inflammation, activated hepatic stellate cells and increased expression of pro-inflammatory and pro-fibrotic genes. As expected, insulin treatment in this model had beneficial effects on glucose control and dyslipidemia and increased body weight and fat mass, but also significantly improved liver histopathology by decreasing hepatic steatosis, inflammation and stellate cell activation. Furthermore, insulin treatment decreased the hepatic expression of pro-inflammatory and pro-fibrotic genes.

In humans, T2DM is an important risk factor for development of NAFLD and NASH and is also an independent predictor of advanced liver fibrosis and liver-related mortality [3–6]. In T2DM, hyperglycemia as well as insulin resistance and hyperinsulinemia could all likely contribute to acceleration of liver pathology, however, their individual impact on NAFLD progression in humans is not clear [43]. In the NASH-STZ hamster we do not expect hyperinsulinemia to be a contributing factor, due to the method of diabetes induction (low-dose STZ), however, because of the prolonged hyperglycemia we do expect the NASH-STZ hamster to be insulin resistant as a consequence of glucose toxicity [44]. Insulin resistance was likely also present in the NASH group, where the levels of endogenous insulin were almost twice as high as in the CTRL group (although with the present groups sizes this difference did not reach significance). However, the NASH group did not have progressive liver disease. This suggests that mild insulin resistance did not have a major influence on liver pathology in NASH hamsters and indicates that hyperglycemia, in the absence of hyperinsulinemia, significantly accelerated development of NAFLD in the NASH-STZ hamster.

Hyperglycemia may have accelerated liver pathology in this model by both direct and indirect mechanisms. High levels of circulating glucose could enhance development of hepatic steatosis by increasing substrate availability for hepatic de novo lipogenesis but also by directly inducing expression of carbohydrate response element-binding protein, which is a transcription factor highly capable of activating lipogenic genes in the liver [45]. Furthermore, hypoinsulinemia in the NASH-STZ hamster could likely have increased peripheral lipolysis due to the lack of insulin-mediated lipase inhibition [20], which in turn results in increased flux of FFA to the liver. In fact, we observed increased levels of plasma FFAs in NASH-STZ hamsters and a significant reduction in mass of the epididymal fat depot, which may indicate increased peripheral lipolysis.

Hyperglycemia has also been shown to possess pro-inflammatory properties in clinical [46–48] and preclinical studies [25–27]. A 75 g oral glucose load as well as hyperglycemic clamping in healthy human subjects has been shown to induce marked inflammatory changes and oxidative stress with formation of reactive oxygen species (ROS) [46–49]. These effects occur also when endogenous insulin secretion is suppressed [47], which to some extent resembles the STZ-induced decrease in the number of functional pancreatic β-cells in our model. ROS has been shown to activate the NF-κB- pathway [50], one of the key inflammatory pathways in NASH [51]. In accordance with this, the transcription factor NF-κB (denoted as “*Rel”* in Additional file 2: Table S2) and several of the inflammatory genes it induces (e.g. *Il6, Ccl2, Ccr2, Il1b, Vcam*) was upregulated in the NASH-STZ-hamster compared to the NASH group. Also, circulating levels of haptoglobin, a suggested biomarker for hepatocyte ballooning in human NASH [52], were increased and hepatic area fractions of CD68+ , CD11b+ and CD45+ cells were all elevated in hyperglycemic animals. The significant increase in amounts of CD68+ cells and expression levels of *Cd68* in the livers of NASH-STZ hamsters indicated marked proliferation of resident macrophages (Kupffer cells) and/or recruitment of mononuclear cells. In human NASH, accumulation of CD68+ cells in the liver increase with disease stage [53] and macrophages (in particular Kupffer cells) play an important role in the progression towards NASH and present potential pharmacological targets [54, 55]. The STZ-NASH hamster model could therefore potentially be valuable for in evaluation of novel drugs aimed at such targets.

Fibrosis is an important prognostic marker in human NASH [56], but our hamster model only displayed minimal perisinusoidal fibrosis. However, the area fraction of hepatic α-SMA + cells and expression of genes involved in hepatic stellate cell activation (*Acta2* (α-SMA), *Col1a1, Col1a2, Col3a1, Col5a3, Col6a1, Mmp9, Timp1* and *Timp2)* and fibrosis (*Col1a1, Col3a1, Timp1, Fap*) were increased in the hyperglycemic STZ-NASH hamsters. These findings indicate that fibrotic processes are initiated but not yet fully manifested within the relatively short study period of 9 weeks. In NASH hepatic stellate cells are activated and transformed into myofibroblast-like cells, which then contribute to the fibrogenic processes [57, 58]. Transformation of hepatic stellate cells can be stimulated by high glucose concentrations in vitro [59, 60]. Furthermore, expression of connective tissue growth factor (CTGF) was significantly increased in the NASH-STZ hamster compared to NASH animals (Additional file 1: Table S4). CTGF is involved in liver fibrosis in both humans and animal models [61–63], and hyperglycemia has been shown to increase mRNA-levels of CTGF in vivo and in vitro [63]. The absence of advanced fibrosis in our model was therefore likely due to the limited length of the diet- and diabetes-induction periods. More advanced stages of fibrosis may develop if these periods were prolonged. Longer diet- and diabetes induction periods may also result in ballooning of hepatocytes, which is an important hallmark of human NASH [64].

In this study, insulin treatment significantly decreased hepatic triglyceride levels with ~ 30%. This is in good agreement with results from several clinical studies in T2DM patients [12–19]. The effects of insulin on liver fat have been suggested to be in part due to inhibition of peripheral lipolysis, which decreases the flux of FFAs to the liver [17]. Although insulin is paradoxically also a potent inducer of hepatic de novo lipogenesis [65], the relative contribution of peripheral lipolysis to liver fat accumulation in insulin-resistant patients with NAFLD has been estimated to exceed that of de novo lipogenesis [66]. We observed a nonsignificant trend towards lower plasma FFA levels in insulin-treated animals, which could explain the decrease in hepatic fat content. The indirect insulin-induced reduction in hepatic lipid content observed in our study and in clinical trials might be theorized to reduce or prevent progression of both inflammation and fibrosis in the liver. However, retrospective studies addressing insulins effect on progression of NAFLD in diabetic patients have reported conflicting results. One study showed insulin treatment to be associated with advanced liver fibrosis [21], whereas another study associated insulin treatment with improvements in liver fibrosis [67]. In addition, insulin treatment has retrospectively been associated with hepatocellular cancer [22, 23]. However, insulin treatment in the NASH-STZ-HI hamsters clearly prevented or reversed liver pathology to the level seen in NASH hamsters, by decreasing hepatic area fractions of CD68+ , CD45+ and CD11b+ cells and hepatic stellate cell activation. In addition, hepatic expression of a wide range of genes involved in inflammation and fibrosis such as *Col3a1* and *Timp1* was decreased. In human NASH, expression of *Col3a1* has been suggested as a biomarker for discrimination between fibrotic and non-fibrotic liver, and has been shown to be positively correlated with liver inflammation activity [68]. *Timp1* is involved in regulation of extracellular matrix remodelling and has been shown to be increased in fibrotic rat and human liver [69, 70]. The top 10 genes significantly upregulated by insulin treatment, were predominantly involved in glucose- (*Pppr1rb3, Gck, Pklr*) and fat metabolism (*Me1, Mogat2, Pnpla3*). *Pppr1rb3* and *Gck* are both involved in regulation of glycogen synthesis [71, 72], in good agreement with the increased amount of liver glycogen observed in insulin-treated animals compared to the hyperglycemic NASH-STZ hamsters. *Me1, Mogat2*, and *Pnpla3* are involved in steps relating to fatty acid or triglyceride metabolism and may in part reflect the stimulatory effects of insulin on transcription factor SREBP1-c [73, 74]. The upregulation of these genes was generally mirrored by a downregulation in the NASH-STZ-hamster, likely reflecting restored insulin-signalling. However, in addition to these—perhaps not unexpected—effects of insulin on gene expression, the list of top 10 genes downregulated by insulin treatment revealed decreased expression of *Tff3, Gpnmb, Soat1* and *Spp1.* Expression of these four genes were all increased in the NASH-STZ- hamster and increased expression of these genes has been implicated in progressive liver disease such as fibrosis and hepatocellular carcinoma [75–83], which further indicates a beneficial effect of insulin treatment on progressive liver disease.

Previous experimental studies addressing the effects of insulin treatment on hepatic inflammation, oxidative stress and fibrosis in diabetic animal models of NAFLD, have yielded varying outcomes [24–27]. In C57BL/6 mice fed a high-fat diet and subsequently injected with STZ to induce diabetes, daily s.c. injections with human insulin for 4 weeks decreased hepatic collagen deposition and expression of *Timp1* but did not show effect on liver macrophage count [24]. In contrast, treatment with insulin detemir for 6 days in STZ-induced hyperglycemic male dYY mice fed a choline-deficient high-fat diet decreased hepatic *Tnfα* expression but did not influence *Col1a1* expression [25]. In a study with STZ-induced hyperglycemic rats fed a chow diet, insulin was administered via osmotic minipumps for 4 weeks, similar to our study. However, glycemic control in the insulin-treated rats was rather poor, and insulin treatment did not decrease systemic and hepatic inflammation as measured by plasma α-2-macroglobuline and hepatic macrophage infiltration [26]. In another study comparing the effects of pump delivered and injected insulin in STZ-induced hyperglycemic rats treated for 4 weeks [27], the glycemic control of the pump-treated group was superior to that demonstrated in [26]. In this study hepatic histological analysis showed reduction in both oxidative stress and inflammation. Interestingly, the effects of insulin on hepatic steatosis were either only briefly mentioned or not reported in the experimental studies described above. Discrepancies in outcomes between those and the present study may be attributed to both differences in species, diet, treatment duration, method of insulin administration and/or differences in glycaemic control. In our study, it is a strength that insulin was administered by constant s.c. infusion using osmotic minipumps, mimicking basal insulin treatment in a well-regulated diabetic patient, without large fluctuations in blood glucose. The obtained glycaemic control was markedly better than in previous studies with insulin treatment in mouse and rat models [24–26]. Furthermore, the beneficial effects of insulin on liver histopathology observed in our study were confirmed at gene expression level, with decreased expression of genes involved in inflammation and fibrosis, which further strengthens the validity of our findings.

The way insulin treatment improved liver pathology and hepatic gene expression profile in this study is likely tightly related to the overall improvement of glycemic and metabolic status. However, insulin could potentially also exert more direct effects on inflammatory cells and hepatic gene expression. Insulin treatment has been shown to lower serum concentrations of IL-6 and high-sensitive C-reactive protein (hsCRP) in newly diagnosed T2DM patients, independently of its blood glucose lowering properties [84]. Also, a 6-h insulin-infusion in combination with 5% dextran infusion in obese, non-diabetic individuals, showed acute suppressive actions of insulin on intranuclear NF-κB and ROS generation in mononuclear cells, with a concurrent decrease in plasma MCP-l (*Ccl2*) [85]. Supporting these findings, insulin treatment decreased hepatic expression levels of NF-κB (*Rel*), *Il*-*6* and *Ccl2* in the NASH-STZ-HI hamster.

On a translational level, the expression patterns of genes that were regulated by more than ± 1.5 fold between human NAFL and NASH patients and were present in the hamster data set, overlapped to a large extent with the expression patterns between the NASH and NASH-STZ hamsters. Thus, even though the NASH-STZ hamsters histologically had not developed NASH (only very mild perisinusoidal fibrosis and no ballooning was observed) the transcriptome of NASH-STZ hamsters to some extent resembled the changes in gene expression observed in human NASH. Further supporting a NASH-like transcriptomic phenotype, several NAFLD candidate genes involved in both inflammatory and fibrotic signaling pathways [39] were significantly upregulated in NASH-STZ hamsters compared to NASH hamsters. However, there were also differences in gene expression between our hamster model and the two human datasets. This is expected when different species and different studies are compared. Furthermore, while we took care in selecting human datasets which we believe were best suited for comparison to the present data from hamsters, it should be remembered that no gold standard human NASH gene expression dataset exists. In the study by Suppli et al., it is for example surprising that expression of several genes associated with monocyte recruitment and inflammation such as *Ccr1, Cd14, Cd68, Cd86, Col6a2* and *Tlr4* were not upregulated in NASH vs NAFL, although these genes have been implicated in progression of NASH [86–90] and were also upregulated in NASH-STZ hamsters. Furthermore, the transcriptome of NAFL and NASH patients [39] was overlapping to a large extent, which was confirmed by histological data, with NASH patients displaying rather mild pathological changes, whereas the NAFL patients displayed not only hepatic steatosis but also mild hepatic inflammation. Also, in the study by Suppli et al., NAFL or NASH patients with confirmed diabetes were excluded. Finally, the hamster is considered a non-model organism, with the genome still only being available on a scaffold level (MesAur1.0, Ensembl Genome Browser). As such, inclusion of genes which currently still lacks annotation could potentially increase the overlap of genes present in the hamster data set and expression data from the two human data sets [39, 40].

The present study design did not allow us to determine whether the beneficial effects of insulin on liver pathology were caused by indirect effects of the restoration of glucose and lipid dysregulation or by direct effects at a cellular or molecular level. However, this would be interesting to address in future studies, possibly including other blood-glucose lowering therapies. Also, since this model is based on a relatively short-term induction- and treatment period (4 weeks on NASH-diet, 1 week for STZ-induction of hyperglycemia, followed by additional 4 weeks treatment with insulin or vehicle), it would be interesting to evaluate how the induced phenotype progress during a longer time span, and also if the treatment effects are caused by prevention or reversal of progressive liver disease. Furthermore, the individual contributions from dietary fat, fructose and cholesterol to the observed phenotype would be relevant to explore in future studies. Due to the marked hepatic steatosis accompanied by significant macrophage infiltration and hepatic stellate cell activation and because all features were achieved within a relatively short time span, the NASH-STZ hamster could be of value in investigating effects of drugs targeting these pathological aspects of NASH under hyperglycemic conditions. In addition, the enrichment and upregulation of pathways and genes involved in inflammation and fibrosis could provide insight into drug effects on transcriptional regulation related to these pathological traits.

## Conclusion

Our results suggest that hyperglycemia is important for development of inflammation and profibrotic processes in the liver, and we show here that insulin administration has beneficial effects on liver pathology and hepatic gene expression in a hyperglycemic, dyslipidemic hamster model of NAFLD. The effect of insulin treatment could be mediated via a direct effect of insulin on inflammatory cells or through an indirect effect of normalization of blood glucose.

## Supplementary Information

**Additional file 1: Table S1.** Immunohistochemistry protocols. Overview of the immunohistochemistry protocols used in the study. **Table S3.** Gene expression in NASH vs CTRL hamsters. Fold changes and log2-fold changes in gene expression between NASH and CTRL hamsters. **Table S4.** Gene expression in NASH-STZ vs NASH hamsters. Fold changes and log2-fold changes in gene expression between NASH-STZ and NASH hamsters. **Table S5.** Gene expression in NASH-STZ-HI vs NASH-STZ hamsters. Fold changes and log2-fold changes in gene expression between NASH-STZ-HI and NASH-STZ hamsters. **Table S6.** Overview of genes significantly regulated in all three data sets (two human data sets and one hamster data set). List of genes exhibiting similar (and significant) gene regulation in both the human data sets used as well as the hamster data set.

**Additional file 2. Table S2.** Regulation of genes in enriched pathways. Overview of regulated genes in enriched pathways.

**Additional file 3: Figure S1.** Regulation of genes in NASH-STZ hamsters and human NASH patients. Heat map of 209 genes differentially regulated between human NAFL (simple steatosis) and NASH patients (Lake et al., 2011, [40], right panel) compared to the same genes in NASH-fed hamsters and NASH-STZ hamsters (left panel). Of the 209 genes, 190 genes showed similar expression patterns in the two comparisons.

## Data Availability

The datasets used and/or analyzed during the current study are available from the corresponding author on reasonable request.

## Abbreviations

ALT: Alanine aminotransferase
AST: Aspartate aminotransferase
FFA: Free fatty acids
HDL: High-density lipoprotein
H&E: Haematoxylin and Eosin
IHC: Immunohistochemistry
LDL: Low-density lipoprotein
NAFLD: Non-alcoholic fatty liver disease
NASH: Non-alcoholic steatohepatitis
RIIE: Rat insulin immunoactivity equivalent
RIN: RNA integrity number
STZ: Streptozotocin
T2DM: Type 2 diabetes mellitus
VLDL: Very-low-density lipoprotein
qMR: Quantitative magnetic resonance
