# Supplementary material for: Insulin treatment improves liver histopathology and decreases expression of inflammatory and fibrogenic genes in a hyperglycemic, dyslipidemic hamster model of NAFLD
Source: J Transl Med. 2021 Feb 17;19:80. doi: 10.1186/s12967-021-02729-1 (PMC7890970; doi:10.1186/s12967-021-02729-1)
Supplement: Supplementary file 2 — Additional file 2. Table S2. Regulation of genes in enriched pathways. Overview of regulated genes in enriched pathways. [file 12967_2021_2729_MOESM2_ESM.docx]

**Additional table 2. Regulation of genes in enriched pathways**

**NASH vs CTRL**

| *Top 10 regulated pathways* | *FDR* | *Significantly regulated genes in pathway (red: upregulation; blue: downregulation)* |
| --- | --- | --- |
| SCAP/SREBP Transcriptional control of cholesterol and fatty acid biosynthesis | 4.87xE-09 | *Srebf1, Fads2, Scap, Srebf2, Lss, Mvk, Dhcr7, Fdps, Insig1, Idi1 Hmgcs2, Hmgcs1, Hmdh, Erg1* |
| Protein folding and maturation_Bradykinin/Kallidin maturation | 2.05xE-06 | *Ace, Enpep, Xpnpep2, Cpb2, Cpn1, Plg, Kng2, Klk10, Klk11, Klk12, Klk13, Klk14, Klk15, Klk1b1, Klk1b24, Klk1b27, Klk1b3, Klk4, Klk5, Klk6, Klk7, Klk8, Klk9, Ace2* |
| Colorectal cancer | 5.59xE-06 | *Wnt6, Egfr, Ephb1, Ephb2, Ephb3, Ephb4, Ephb6, Igf1, Met, Tnfrsf1a, Il6st, Dll1, Lepr, Il6, Hgf, Cxcr2* |
| Immune response_IL-6-induced acute-phase response in hepatocytes | 5.59xE-06 | *Serpine1, Kng1, C3, Il6st, Stat3, Crp, Cebpd, Il6, Apcs, Fga, Fgg, Fgb, Saa3* |
| Immune response_ Lectin-induced complement pathway | 6.39xE-06 | *Mbl2, Cd46, C4b, C4a, C8a, C3, Masp1, Cfi, Masp2* |
| Blood coagulation | 1.13xE-05 | *Serpine1, Cpb2, Plg, Kng2, Serpinf2, F3, F8, Fga, Fgg, Fgb,* |
| Putative pathways of activation of classical complement system in major depressive disorder | 1.50xE-05 | *Il6, C1qa, C1qb, C4a, C4b, C4b, C3, C3b, C1qc, C1qa* |
| Cell cycle_the metaphase checkpoint | 2.80xE-05 | *Spc25, Zwint, Cdc20, Bub1, Cenph, Cenpe, Zwilch, Spc24, Dsn1, Nek2, Mad2l1, Aurka* |
| PI3K signaling in gastric cancer | 3.60xE-05 | *Spp1, Nrg1, Gna11, Gna15, Gnaq, Serpine1, Ccnd1, Prnp, Akt1, Akt2, Akt3, Irs1, Egfr, Met, Bmp2, Chuk, Hgf, Cxcr2* |
| Role of IL-6 in obesity and type 2 diabetes in adipocytes | 4.55xE-05 | *Serpine1, Cpt1A, Prkcd, Irs1, Il6st, Ppargc1a, Stat3, Crp, Il6, Socs3, Saa3* |

**NASH-STZ vs NASH**

| *Top 10 regulated pathways* | *FDR* | *Significantly regulated genes in pathway (red: upregulation; blue: downregulation)* |
| --- | --- | --- |
| Macrophage and dendritic cell phenotype shift in cancer | 1.79xE-12 | *Il6, Il12b, Postn, Cd40lg, Ptger2, Cd86, Tlr2, Tlr7, Tlr4, Msr1, Rela, Nos2, Rel, Irf5, Cd40, Cd80, Inpp5d, Thbs1, Cxcl10, Socs3, Il1b, Socs1, Nfkbie, Mertk, Csf1r, H2-Aa, H2-Ab1, H2-DMa, H2-DMb1, H2-Ea-ps, H2-Eb1, H2-Eb2, Tgfb1, Wnt5a, Tnf, Ido1, Pgf, Csf1, Ptger4, Mfge8, Tgfbr1, Rel, Irf4, Notch1,Jag1, Ifngr1, Tnfsf10, Stat6, Esr1, Inhba* |
| Cell adhesion_ECM remodelling | 2.36xE-11 | *Mmp9, Igf2, Plau, Serpine1, Mmp12, Klk1b1, Klk1b22, Klk1b3, Timp1, Cd44, Mmp16, Mmp2, Col1a2, Timp2, Msn, Col4a6, Col3a1, Timp3, Mmp14, Serpine2, Sparc, Lama4, Nid1, Igf1R, Ezr, Vtn, Plg, Fn1, Igf1* |
| Cell adhesion_Integrin inside-out signaling in neutrophils | 2.36xE-11 | *Sell, Sele, Pip5k1a, Pip5k1b, Pip5k1b, Gng2, Cd44, Pik3r5, Ptafr, Tyrobp, Ager, Selplg, Hck, Nos2, Syk, Prex1, Btk, Lcp2, Fermt3, Apbb1ip, Plcb2, Cxcr2, Selp, Itpr1, Itpr2, Itpr3, Fyb, Fgr, Fpr1, Vasp, Pld2, Lyn, Gnai2, Gnai1, Gnai3, Gnao1, Gnaz, Cyth1, Rasgrp2, Pik3cg, Prkcd, Prkg1, Prkg2* |
| Stromal-epithelial interaction in prostate cancer | 1.12xE-11 | *Il6, Mmp9, Timp1, Mmp2, Tnc, Col1a2, Vim, Timp2, Tgfb3, Tgfb1, Snai1, Hgf, Met, Pdgfrb, Pdgfb, Pdgfa, Tgfb2, Igf1r, Fgfr1, Tgfbr1, Jun, Krt18, Fn1, Igf1, Ar* |
| Chemokines in inflammation in adipose tissue and liver in obesity, type 2 diabetes and metabolic syndrome X | 5.25xE-09 | *Il6, Sell, Cxcr4, Cd68, Ccl2, Sele, Cd86, Tlr2, Mrc1, Tlr4, Cd44, Selplg, Itgal, Il1b, H2-Aa, H2-Ab1, H2-DMa, H2-DMb1, H2-Ea-ps, H2-Eb1, H2-Eb2, Itgax, Tnf, Cd14, Cd34, Selp, Ccr2, Vcam1, Pecam1, Itga4, Cd163* |
| Role of fibroblasts in the sensitization phase of allergic contact dermatitis | 1.50xE-08 | *Il6, Mmp9, Cxcr4, Mmp2, Col1a2, Rel, Fos, Atf2, Fosb, Fosl1, Fosl2, Jun, Junb, Jund, Eln, Il1b, Col4a6, Col3a1, Tnf, Il1a, Itga6, Cxcll2, Fn1* |
| IL-1β and Endothelin-1-induced fibroblast/myofibroblast migration and extracellular matrix production in asthmatic airways | 1.50xE-08 | *Ccl2, Serpine1, Timp1, Mmp2, Col1a2, Col1a1, Rel, Thbs1, Fos, Atf2, Fosb, Fosl1, Fosl2, Junb, Jund Il1b, Col3a1, Timp3, Dcn, Col1a2, Ednrb, Il1a, Col4a1, Pdgfrb, Jun, Fn1, Abcc5* |
| Production and activation of TGF-beta in airway smooth muscle cells | 3.63xE-08 | *Plau, Elane, Serpine1, Tlr4, Egr1, F2rl1, Rela, Fos, Atf2, Fosb, Fosl1, Fosl2, Jun, Junb, Jund, Tgfb3, Tgfb1, Gnai2, Gnai1, Gnai3, Gnao1, Gnaz, Tgfb2, Tgfbr1, Nfkbia, Plg* |
| TGF-beta-induced fibroblast/myofibroblast migration and extracellular matrix production in asthmatic airways | 3.63xE-08 | *Mmp9, Serpine1, Timp1, Mmp2, Tnc, Col1a2, Col1a1, Thbs2, Timp2, Fos, Atf2, Fosb, Fosl1, Fosl2, Jun, Junb, Jund, Tgfb3, Col4a6, Pik3ca, Pik3cb, Pik3cd, Col3a1, Tgfb1, Timp3, Dcn, Col1a2, Col4a1, Tgfb2, Col5a1, Tgfbr1, Bgn, Prkcd, Fn1, Abcc5* |
| Renal tubulointerstitial injury in Lupus Nephritis | 5.08xE-08 | *Il6, Plau, Ccl2, Cd40lg, Serpine1, Cd44, Col1a2, Rela, Cd40, Vim, Tnfrsf13c, Il1b, Socs1, Nfkbie, Col3a1, Cd4, Csf1r, H2-Aa, H2-Ab1, H2-DMa, H2-DMb1, H2-Ea-ps, H2-Eb1, H2-Eb2, Tgfb1, Tnf, Tnfsf13b, Tnfrsf1b, Ccr2, Chuk, Csf1, Vcam1, Fn1, Cxcr5* |

**NASH-STZ-HI vs NASH-STZ**

| *Top 10 regulated pathways* | *FDR* | *Significantly regulated genes in pathway (red: up-regulated; blue: down-regulated)* |
| --- | --- | --- |
| Cell adhesion_Integrin inside-out-signaling in neutrophils | 6.53xE-12 | *Sell, PIP5KI, Ager, Ptafr, Pip5k1a, Pip5k1b, Pip5k1c, Cd44, Tyrobp, Selplg, Btk, Cxcr2, Syk, Prex1, Hck, Lcp2, Selp, Fermt3, Apbb1ip, Plcb2, Fyb, Itpr1, Itpr2, Itpr3, Fpr1, Fgr, Gng2, Lyn, Pik3r5, Pik3cg, Vasp, Gnai2, Gnai2, Gnai1, Gnai3, Gnao1, Gnaz, Pld2, Prkcd* |
| Cell adhesion_ECM remodeling | 8.02xE-09 | *Mmp7, Mmp9, Plau, Serpine1, Mmp12, Timp1, Cd44, Klk1b3, Klk1b22, Klk1b1, Timp2, Mmp2, Msn, Mmp14, Col4a6, Col3a1, Lama4, Timp3, Nid1, Igf1r, Sparc, Serpine2* |
| Macrophage and dendritic cell phenotype shift in cancer | 2.64xE-08 | *Ptger2, Postn, Msr1, Tlr4, Cd86, Tlr7, Cd40, Cd80, Wnt5a, Inpp5d, Thbs1, Mertk, Irf5, Il1b, Csf1r, Cxcl10, Tgfb1, Ifngr1, Nfkbie, Il6, Pgf, Csf1, H2-Aa, H2-Ab1, H2-DMa, H2-DMb1, H2-Ea-ps, H2-Eb1, H2-Eb2, Tgfbr1, Rel, Nfkb1, Esr1, Inhba* |
| Ovarian cancer (main signaling cascades) | 2.64xE-08 | *Mmp9, Plau, Pip5k1a, Pip5k1b, Pip5k1c, Myc, Mmp2, Erbb2, Pik3ca, Pik3cb, Pik3cd, Snai1, Chuk, Nfkbie, Met, Il6, Sos1, Sos2, Hgf, Gng2, Akt1, Akt2, Akt3, Lpar1, Prkar1a, Prkar1b, Prkar2a, Prkar2b, Pik3r5, Rel, Gnai2, Gnai1, Gnai3, Gnao1, Gnaz, Tcf, Esr1* |
| Transcription HIF-1 targets | 6.00xE-07 | *Mmp9, Hk2, Cxcr4, Igfbp1, Lgals1, Serpine1, Myc, Thbs1, Mmp2, Pmaip1, Hmox1. Hk1, Lox, Slc2a1, Tgfb1, Ddit4, Met, Pgf, Tgfb3, Tgm2, Loxl4, Mcl1, P4ha1, Loxl2, Tng, Bhlhe40* |
| Immune respons_C3a signaling | 6.00xE-07 | *Ccl2, Cd86, Pip5k1a, Pip5k1b, Pip5k1c, C3ar1, Cd40, Il1b, Adcy1, Adcy2, Adcy3, Adcy4, Adcy5, Adcy6, Adcy7, Adcy8, Adcy9, Plcb1, Plcb2, Plcb3, Plcb4, Chuk, Tgfb1, Il6, Itpr1, Itpr2, Itpr3, H2-Aa, H2-Ab1, H2-DMa, H2-DMb1, H2-Ea-ps, H2-Eb1, H2-Eb2, Gng2, Akt1, Akt2, Akt3, Klf5, Pik3r5, Gnai2, Gnai1, Gnai3, Gnao1, Gnaz* |
| Chemokines in inflammation in adipose tissue and liver in obesity, type 2 diabetes and metabolic syndrome X | 6.00xE-07 | *Sell, Mrc1, Cd68, Cxcr4, Ccl2, Tlr4, Cd86, Cd44, Selplg, Selp, Il1b, Cd14, Itgal, Il6, Cd34, H2-Aa, H2-Ab1, H2-DMa, H2-DMb1, H2-Ea-ps, H2-Eb1, H2-Eb2, Pecam1, Cd163* |
| Inhibition of neutrophil migration by proresolving lipid mediators in COPD | 6.16xE-07 | *Sell, Tlr4, C5ar1, Ptafr, Pip5k1a, Pip5k1b, Pip5k1c, Cxcr2, Vav1, Prex1, Il1b, Msn, Plcb2, Adam17, Itpr1, Itpr2, Itpr3, Cd34, Fpr1, Gng2, Tnfrsf1b, Pecam1, Pik3r5, Rel, Prkca, Prkcb, Prkcd, Prkce, Prkcg, Prkch, Prkci, Prkcz, Gnai2, Gnai1, Gnai3, Gnao1, Gnaz* |
| CDHI Correlations from Replication data_Causal network (positive correlations) | 9.33xE-07 | *Cxcr4, Pip5k1a, Pip5k1b, Pip5k1c, Cd44, Cd40, Cd80, Lcp2, Ptk2b, Hip1, Cd83, Pik3ca, Pik3cb, Pik3cd, Il1a, Chuk, Nfatc2, Nfkbie, Itpr1, Itpr2, Itpr3, Rps6ka4, Rps6ka5, H2-Aa, H2-Ab1, H2-DMa, H2-DMb1, H2-Ea-ps, H2-Eb1, H2-Eb2, Gng2, Akt1, Akt2, Akt3, Pik3r5, Rel, Casp3, Hspa14, Hspa1a, Hspa1b, Hspa1I, Hspa2, Hspa4, Hspa5, Hspa8, Hspa9* |
| Immune response_PIP3 signaling in B-lymphocytes | 2.46xE-06 | *Pip5k1a, Pip5k1b, Pip5k1c, Btk, Vav1, Syk, Prex1, Inpp5d, Pik3ca, Pik3cb, Pik3cd, Plcb1, Plcb2, Plcb3, Plcb4, Rps6ka1, Rps6ka2, Rps6ka3, Fcgr2b, Dapp1, Itpr1, Itpr2, Itpr3, Gng2, Akt1, Akt2, Akt3, Lyn, Pik3r5* |
