# Supplementary figures and images for: Insulin treatment improves liver histopathology and decreases expression of inflammatory and fibrogenic genes in a hyperglycemic, dyslipidemic hamster model of NAFLD
Source: J Transl Med. 2021 Feb 17;19:80. doi: 10.1186/s12967-021-02729-1 (PMC7890970; doi:10.1186/s12967-021-02729-1)

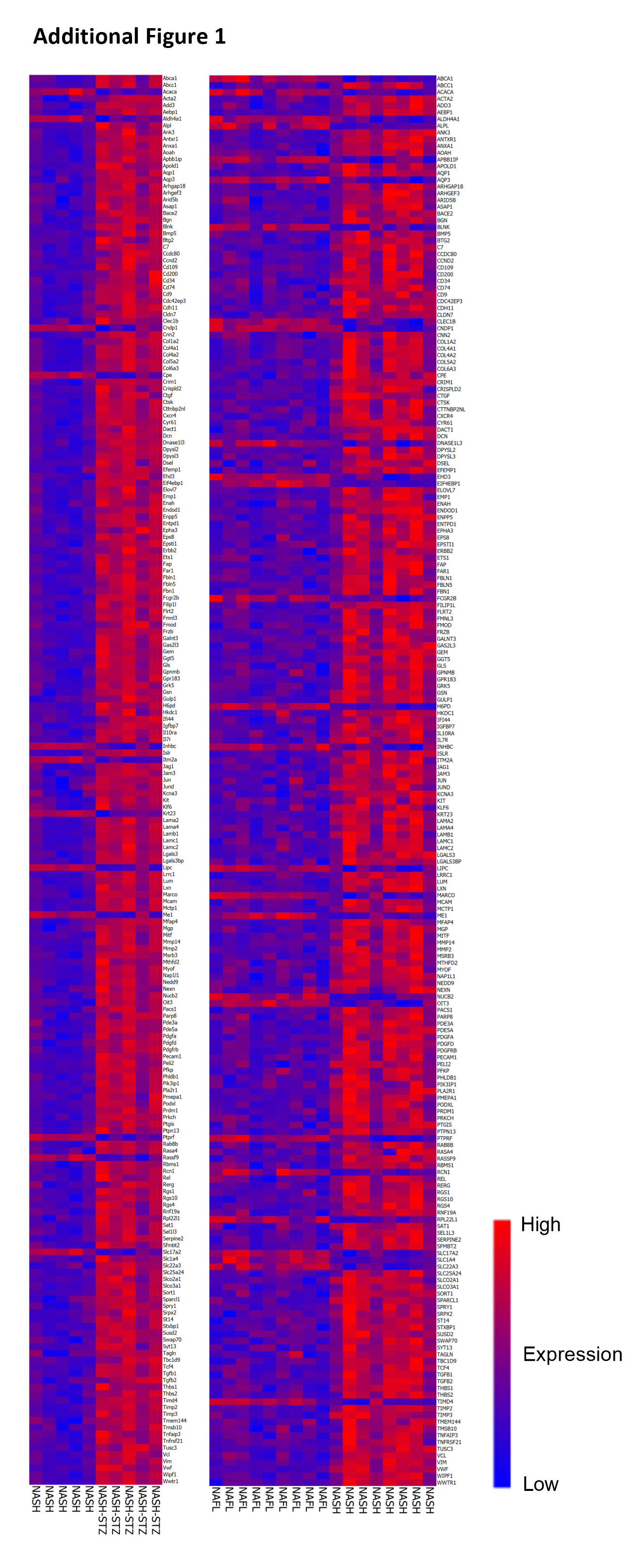

Supplement: Supplementary file 3 — Additional file 3: Figure S1. Regulation of genes in NASH-STZ hamsters and human NASH patients. Heat map of 209 genes differentially regulated between human NAFL (simple steatosis) and NASH patients (Lake et al., 2011, [40], right panel) compared to the same genes in NASH-fed hamsters and NASH-STZ hamsters (left panel). Of the 209 genes, 190 genes showed similar expression patterns in the two comparisons. [file 12967_2021_2729_MOESM3_ESM.tif]
